# In Search of Effective UiO-66 Metal–Organic Frameworks for Artificial Kidney Application

**DOI:** 10.1021/acsami.1c05972

**Published:** 2021-09-14

**Authors:** Klaudia Dymek, Grzegorz Kurowski, Łukasz Kuterasiński, Roman Jędrzejczyk, Magdalena Szumera, Maciej Sitarz, Anna Pajdak, Łukasz Kurach, Anna Boguszewska-Czubara, Przemysław J. Jodłowski

**Affiliations:** † Faculty of Chemical Engineering and Technology, Cracow University of Technology, Warszawska 24, 30-155 Kraków, Poland; ‡ Jerzy Haber Institute of Catalysis and Surface Chemistry, Polish Academy of Sciences, Niezapominajek 8, 30-239 Kraków, Poland; § Małopolska Centre of Biotechnology, Jagiellonian University, ul. Gronostajowa 7A, 30-387 Kraków, Poland; ∥ Faculty of Materials Science and Ceramics, AGH University of Science and Technology, Mickiewicza 30, 30-059 Kraków, Poland; ⊥ Strata Mechanics Research Institute, Polish Academy of Sciences, Reymonta 27, 30-059 Kraków, Poland; # Independent Laboratory of Behavioral Studies, Medical University of Lublin, 4A Chodzki Str., 20-093 Lublin, Poland; ∇ Department of Medical Chemistry, Medical University of Lublin, 4A Chodzki Str., 20-093 Lublin, Poland

**Keywords:** metal−organic frameworks, UiO-66, UiO-66-NH_2_, uremic toxins, adsorption

## Abstract

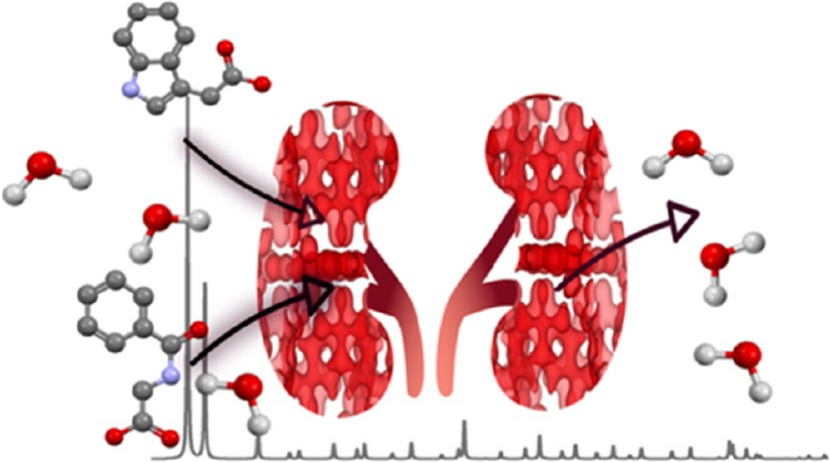

The removal of uremic toxins from patients with acute kidney injury is a key issue in improving the quality of life for people requiring peritoneal dialysis. The currently utilized method for the removal of uremic toxins from the human organism is hemodialysis, performed on semipermeable membranes where the uremic toxins, along with small molecules, are separated from proteins and blood cells. In this study, we describe a mixed-linker modulated synthesis of zirconium-based metal–organic frameworks for efficient removal of uremic toxins. We determined that the efficient adsorption of uremic toxins is achieved by optimizing the ratio between −amino functionalization of the UiO-66 structure with 75% of −NH_2_ groups within organic linker structure. The maximum adsorption of hippuric acid and 3-indoloacetic acid was achieved by UiO-66-NH_2_ (75%) and by UiO-66-NH_2_ (75%) 12.5% HCl prepared by modulated synthesis. Furthermore, UiO-66-NH_2_ (75%) almost completely adsorbs 3-indoloacetic acid bound to bovine serum albumin, which was used as a model protein to which uremic toxins bind in the human body. The high adsorption capacity was confirmed in recyclability test, which showed almost 80% removal of 3-indoloacetic acid after the third adsorption cycle. Furthermore, *in vitro* cytotoxicity tests as well as hemolytic activity assay have proven that the UiO-66-based materials can be considered as potentially safe for hemodialytic purposes in living organisms.

## Introduction

Acute kidney injury (AKI), formerly defined as acute renal failure, is a serious disease in which disorders in the glomerular filtration rate lead to the accumulation of the products of metabolisms in the human body.^1^ The products that would normally be filtered by the kidneys are called uremic toxins.^2^ The groups of uremic toxins consist of a large number of molecules that are divided into three main groups varying in molecular weight—<500 D, ≥500 D, and having the ability of protein binding and water solubility.^2^ The uremic toxins represent a wide variety of molecules from organic groups including ribonucleosides, guanidines, polyols, purines, indoles, and polyamines.^2^ The multitude and diversity of their physicochemical properties require highly effective uremic toxin removal methods to improve the quality of life for end-state renal disease patients (ESRD). Currently, the most commonly used method for the removal of uremic toxins from the organisms of patients with ESRD is hemodialysis (HD). In this process, the patient’s blood passes through semipermeable membranes and the small molecules are separated from proteins and blood cells.^2^ The membranes that are currently in use are made of cellulose/modified cellulose, polyamide, or polyacrylonitrile.^2^ Apart from HD and transplant methods, a wearable artificial kidney option (WAK) is actually under intense investigation.^3^ The advantage of WAK over conventional hemodialysis methods is the minimization of the need for hospital care, resulting in an improvement in the quality of life of people requiring peritoneal dialysis.

Despite the development of complex artificial kidneys that could improve the quality of life of people with severe renal injury, the development of novel adsorbent materials for the efficient removal of uremic toxins is critical to further develop care for patients with AKI.

From among the literature reports on the use of materials such as zeolites,^4^ carbon nanotubes,^5^ ordered mesoporous carbon,^6^ and metal–organic frameworks (MOF),^7−10^ the latter seems to be a relatively unexplored area. Although metal–organic frameworks have been successfully used in various applications including gas adsorption and separation, adsorption of toxins,^11,12^ environmental protection,^13,14^ catalysis,^15−17^ medicine, and drug release,^12,18^ their use as effective adsorbents of uremic toxins is currently undergoing intensive research.^7−10^

Among literature reports, several MOFs have been investigated as potential adsorbents for uremic toxins. In the work by Cuchiaro et al.,^8^ the adsorption efficiency of zirconium-based metal–organic framework MOF-808 and its iron analogue MIL-100(Fe) was tested in adsorption of *p*-cresyl sulfate. The maximum adsorption for MOF-808 and MIL-100(Fe) was 23.6 and 68.6 nmol/mg of MOF, respectively. The greater sorption efficiency of MIL-100(Fe) was hypothetically attributed to the direct coordination of uremic toxins to the vacant metal sites available in the MOF structure. In the work by Kato et al.,^9^ the zirconium-based metal–organic framework represented by NU-1000 was examined in the adsorption of potassium *p*-cresyl sulfate, potassium indoxyl sulfate, and hippuric acid. The maximum sorption of 0.1 mM uremic toxins on 6 mg of NU-1000 was achieved for hippuric acid and potassium indoxyl sulfate after 5 h, whereas for potassium *p*-cresyl sulfate, the maximum sorption after 5 h was equal to 80%.

A high creatinine adsorption capacity was also reported for UiO-66-(COOH)_2_/cotton fabric composite in the work by Abdelhameed et al.^19^ In their work, the UiO-66-(COOH)_2_ was grown directly within cotton fabric. The Zr-based MOF/cotton fabric composite material was tested in the adsorption of creatinine from a Tyrode buffer solution. The prepared material exhibited 113.6 and 192.3–212.8 mg/g sorption capacities for parent UiO-66-(COOH)_2_ and composite UiO-66-(COOH)_2_/cotton fabric, respectively. The UiO-66-(COOH)_2_/cotton fabric materials also exhibited high creatinine adsorption recyclability.

Among the range of potential MOF structures with high adsorption parameters and high chemical resistance, UiO-66 seems to be very attractive due to its high thermal and chemical stability. In addition, the possibility of its modification by the incorporation of functional groups and optimization of the pore size by modulated synthesis makes UiO-66 a potential candidate for use in the sorption of uremic toxins. Recently, the adsorption of potassium *p*-cresyl sulfate, potassium indoxyl sulfate, and hippuric acid was tested in the work by Kato et al.^9^ over pristine UiO-66. The maximum removal efficiencies reached during the sorption of 1.5 mg of UiO-66 in 0.1 mM potassium *p*-cresyl sulfate, potassium indoxyl sulfate or hippuric acid were 2.1, 21, and 90%, respectively. However, it must be emphasized that the structure of the UiO-66 prepared in this work was nearly defect-free, which may considerably decrease the sorption capacity.^18^ Additionally, the modification of pristine UiO-66 by the incorporation of isovalent substituents such as −NH_2_, −OH, and SO_2_H considerably increases sorption capacity by changing the electronic properties of the framework.^20^

**Figure 1:**
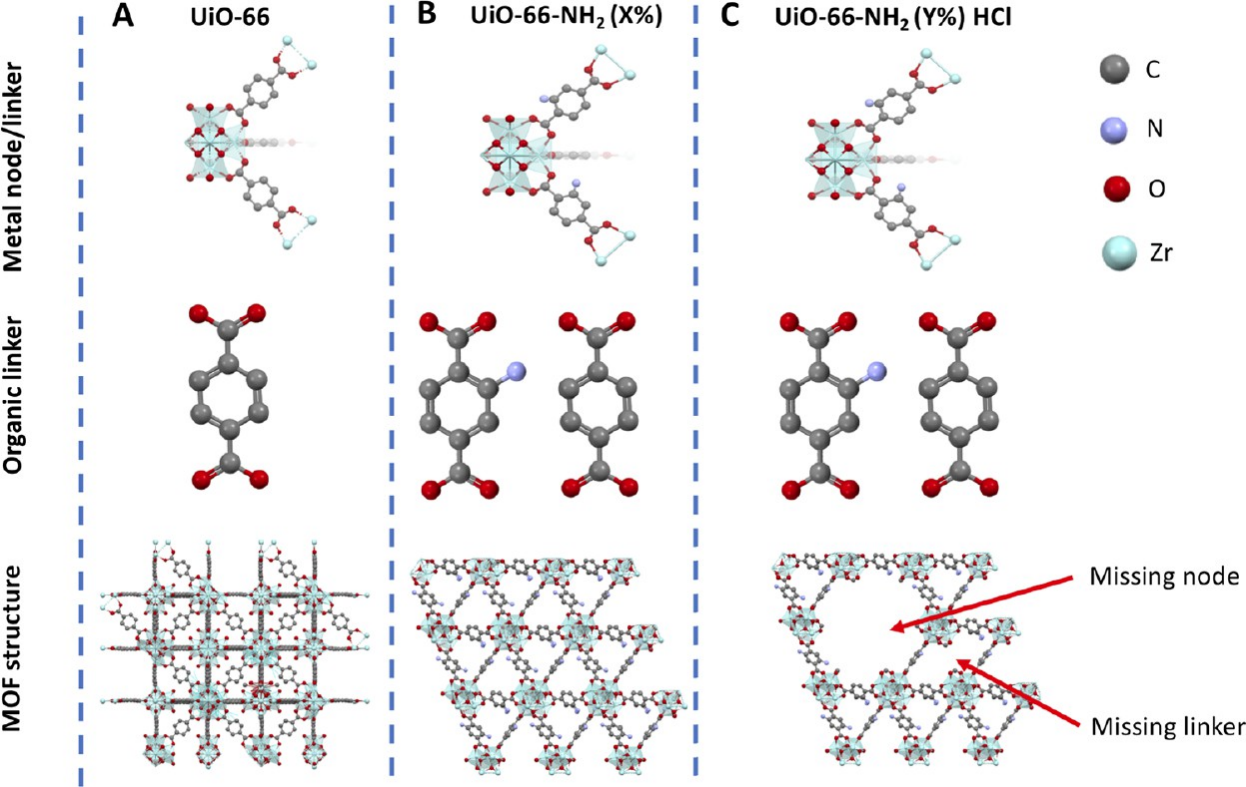
Structures of (A) UiO-66, (B) UiO-66-NH_2_, and (C) defective UiO-66-NH_2_; *X*%—wt % of H2BDC-NH_2_ used; *Y*%—vol % of HCl used during the synthesis.

In this work, we present a systematic approach to obtaining UiO-66 materials with varying concentration of −amino groups in parallel with defect engineering by the addition of concentrated hydrochloric acid during modulated synthesis. Since the preparation of the −amino-functionalized UiO-66 materials has been recently reported in a few papers,^20−22^ the information about the influence of the modulator addition on specific adsorption parameters optimized during the modulated synthesis is rather limited. The schematic representation of the crystal structures of mixed-linker and defective UiO-66 samples in “idealized” form is presented in Figure 1. The mixed-linker UiO-66-NH_2_ crystal structure is presented in Figure 1B. It must be pointed out that despite the fact that the amount of the −amino groups is defined by their amount during the synthesis step, their location in the resulting MOF structure and the sequence of occurrence in the crystal structure is stochastic rather than purely sequential. An additional aim of this paper is to combine both the presence of the specified amounts of −NH_2_ groups and the structural defects shown in idealized form (Figure 1C).

Understanding the adsorption of uremic toxins over metal–organic frameworks to further optimize their sorption properties requires a complementary approach that considers structural and morphological properties. Literature findings do not differentiate between the high surface area of prepared MOF and their electronic properties but rather attempt to rationalize MOF synthesis in both properties.^8^

Taking into consideration economic factors, we would like to focus on the optimization of the synthesis of defective UiO-66 to obtain highly efficient adsorbents of uremic toxins. To achieve that, we present the method of obtaining a series of UiO-66 metal–organic frameworks with optimized morphology by increasing the number of structural defects and electronic properties by functionalizing 1,4-benzene-dicarboxylate linkers by −NH_2_ groups. The complementary approach to the engineering of defective metal–organic frameworks allowed us to obtain materials with high affinity for uremic toxins and low-toxicity profile versus experimental models for epithelium cells and red blood cells (RBCs).

## Experimental Section

The details of material synthesis and activation are described in detail in the Supporting Information. A detailed description of the characterization methods, including PXRD, UV–Vis, DR UV–Vis, DLS, *S*_BET_, dissolution/liquid ^1^H NMR, SEM, *in situ* DRIFT, DSC-TGA, simulation (PXRD and electron densities), *in vivo* and *in vitro* tests, is provided in the Supporting Information. The synthesis details of all prepared samples are summarized in Table S1.

## Results

We synthesized the series of pristine and defective UiO-66 and UiO-66-NH_2_ varying the content of hydrochloric acid used as a modulator and H_2_BDC and H_2_BDC-NH_2_ in mixed-linker synthesis. The PXRD patterns for pristine and defective UiO-66 samples (Figure S1) are in good agreement with the literature data^23,24^ and simulated PXRD patterns^25^ (Figures S1 and S2). Additionally, high-crystallinity materials were obtained for mixed-linker UiO-66 synthesis (Figure S1B), and defective UiO-66-NH_2_ (75%) 25% HCl and UiO-66-NH_2_ (75%) 12.5% HCl showed no difference in PXRD diffractograms in comparison with the pristine UiO-66 sample and defective UiO-66 *X*% HCl (Figure S1A).

Quantitative UV–vis analysis of the −amino-functionalized UiO-66 previously proposed by Chavan et al.^21^ showed that the determined H_2_BDC-NH_2_ content corresponds with theoretical values (Tables 1 and S2, Figure S3). Slight deviations between the experiment and theoretical contents of H_2_BDC-NH_2_ in the prepared samples were observed while increasing the −NH_2_ content in mixed-linker synthesis (UiO-66-NH_2_ (25–100%)) and became considerable in UiO-66-NH_2_ (75%) 25% HCl and UiO-66-NH_2_ (75%) 12.5% HCl samples. We seek the cause of this in the fact that these samples were prepared with a considerable amount of hydrochloric acid (25 and 12.5 vol %) during modulated synthesis of UiO-66-NH_2_ (75%).

**Table 1:** Synthesis Details and Low-Temperature N_2_ Sorption Results

sample	theoretical mol % of H_2_BDC-NH_2_	exp. mol % of H_2_BDC-NH_2_	*S*_BET_, m^2^/g	*S*_Lang._, m^2^/g	*V*_micro_^a^, cm^3^/g	*D*^b^, Å	particle size^c^, nm
UiO-66	n.a.	n.a.	1072.8	1328.8	0.545	19.23	532.4 ± 0.2
UiO-66 12.5% HCl	n.a.	n.a.	1132.6	1496.1	0.568	23.26	378.8 ± 3.3
UiO-66 25% HCl	n.a.	n.a.	1241.5	1547.0	0.626	23.87	291.6 ± 11.7
UiO-66-NH_2_ (25%)	25	32	993.6	1228.7	0.487	19.49	802.6 ± 27.9
UiO-66-NH_2_ (50%)	50	49	936.4	1142.4	0.443	19.60	843.1 ± 38.5
UiO-66-NH_2_ (75%)	75	68	929.9	1136.0	0.465	19.21	608.5 ± 18.7
UiO-66-NH_2_ (100%)	100	82	801.4	933.6	0.397	18.88	515.1 ± 3.9
UiO-66-NH_2_ (75%) 12.5% HCl	75	71	947.4	1124.1	0.484	19.04	414.7 ± 7.5
UiO-66-NH_2_ (75%) 25% HCl	75	52	1062.5	1208.5	0.512	18.77	313.3 ± 4.7

a Total pore volume calculated from the NLDFT model.

b Average pore diameter calculated from the BET model.

c Particle size determined from the DLS analysis.

To investigate the influence of the synthesis parameters on the structural properties, low-temperature nitrogen adsorption isotherms were performed and corresponding BET and Langmuir surface areas were calculated (Figure S4, Table 1). The BET surface area of pristine UiO-66 was 1073 m^2^/g with pore diameters of 5–9 and 14–18 Å (cf. Figure S4). As expected, defective UiO-66 showed an increased sorption capacity. The addition of HCl caused the opening of a new free pore space in the material, which resulted in an increase in their specific surface area values. The considerable increase of SSA_BET_ from 1073 m^2^/g (pristine UiO-66) to 1133 m^2^/g for UiO-66 12.5% HCl and to 1242 m^2^/g for UiO-66 25% HCl was determined. Additional pores with diameters in the range of 14–20 Å were opened for UiO-66 12.5% HCl and 5–8 and 17–22 Å for UiO-66 25% HCl, respectively.

The functionalized UiO-66 with amine groups showed the reduction of nitrogen sorption capacity, which was caused by part of their previously free pore space. As the content of the −amino groups increased, the SSA_BET_ value decreased, and for the 100% substitution of H_2_BDC by H_2_BDC-NH_2_, the SSA_BET_ was equal to 801 m^2^/g. We observed ambiguous changes in the pore size distribution and a decrease in total pore volume from 0.545 cm^3^/g (UiO-66) to 0.397 cm^3^/g (UiO-66-NH_2_ (100%)), which is in good agreement with the literature data.^21,26,27^ The value of the average pore diameter in the defected samples decreased slightly with increasing NH_2_ percentage concentration.

The thermogravimetric analysis–differential scanning calorimetry (TGA/DSC) results are presented in Figure S5. The obtained TGA/DSC curves represent the characteristic shape for UiO-66 samples revealing a few percent mass loss at temperatures of approximately 100 °C associated with the removal of physisorbed water. The mass loss proceeds up to a temperature of approximately 250 °C, where zirconium oxo-clusters are dehydroxylated.^28^ A considerable mass loss is observed for all samples at approximately 350 °C and at 430–490 °C, which is associated with the decomposition of formate ligands^28^ (approximately 350 °C) and H_2_BDC-NH_2_ (430 °C) and H_2_BDC (430 °C),^21^ respectively. The TGA/DSC for defective UiO-66 samples (Figure S5)—UiO-66, UiO-66 12.5% HCl, and UiO-66 25% HCl—shows similar trends in the TGA profile, although the defective UiO-66 25% HCl is considerably linker-deficient compared to the UiO-66 12.5% HCl and UiO-66 samples, respectively.

The molecular structure of the prepared samples was determined by spectroscopic techniques including *in situ* DRIFT spectroscopy (Figure S6), dissolution ^1^H NMR spectroscopy (Figures S7 and S8), and DR UV–Vis spectroscopy (Figure S9). The *in situ* DRIFT spectra for the parent for all prepared samples reveal a characteristic UiO-66 fingerprint in the 1750–650 cm^–1^ region with characteristic bands from the organic linker (Figure S6). The UiO-66-NH_2_ samples prepared via the mixed-linker strategy reveal additional bands at approximately 3469 and 3386 cm^–1^ corresponding to asymmetric and symmetric −NH_2_ stretching modes (Figure S6B). The intensity of those bands increases gradually with increasing H_2_BDC-NH_2_ content used during the mixed-linker synthesis. The DRIFT spectra of defective UiO-66-NH_2_ (75%) 25% HCl and UiO-66-NH_2_ (75%) 12.5% HCl do not exhibit differences between their counterparts prepared without the addition of HCl during modulated synthesis.

The precise determination of the molecular structure of prepared samples was performed by the dissolution/liquid ^1^H NMR method previously proposed by Shearer et al.^28^ The proposed method assumes the dissolution of the MOF framework in deuterated digestion medium and therefore identification of the organic linker, solvent, and modulator in prepared samples. The method also allows determination of whether the monocarboxylates compensate the defects in prepared samples (Figures S7 and S8 for as-received and activated samples, and Table S3). The representative spectrum of the pristine UiO-66 sample prepared with the addition of 9.2 mL of acetic acid is presented in Figure S8A with signals from BDC^2–^ (chemical shift 7.73 ppm) and from formate (ca. 8.3 ppm) and acetate groups (ca. 1.84 ppm). The dissolution ^1^H NMR spectra for −amino-substituted samples (Figure S8B,C) reveal additional signals in a range of 7–7.73 ppm from BDC-NH_2_^2–^. It is worth mentioning that, in samples prepared with the addition of concentrated hydrochloric acid, the acetate signals of dissolution/liquid ^1^H NMR were not detected, whereas in all samples, formate was detected in small amounts. Additionally, an increasing intensity of signals in a range of 7–7.73 ppm originating from BDC-NH_2_^2–^ was observed with increasing content of 2-aminoterephthalic acid used during mixed-linker synthesis with a simultaneous decrease of intensity of BDC^2–^ originating from terephthalic acid. The BDC^2–^ signal disappears completely from UiO-66-NH_2_ (100%), where only 2-aminoterephthalic acid was used during mixed-linker synthesis.

**Figure 2:**
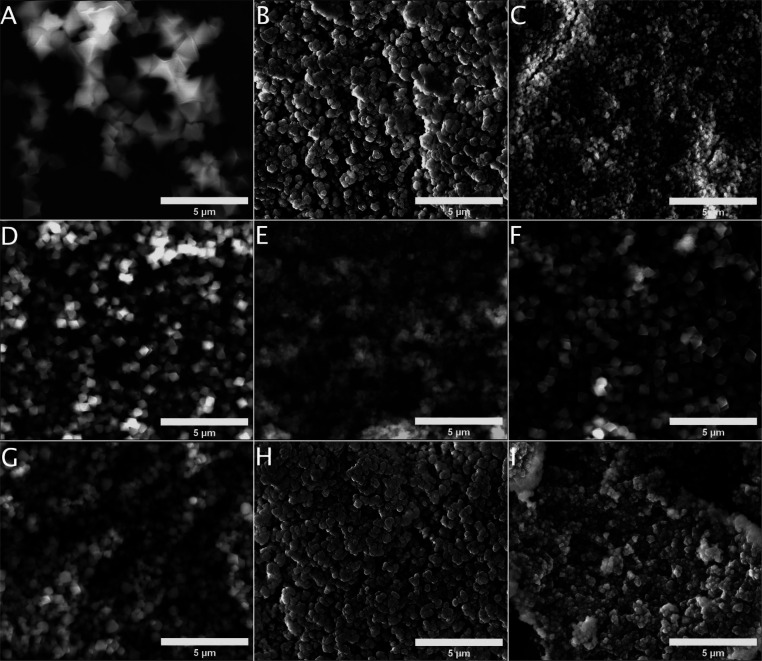
SEM micrographs of prepared samples: (A) UiO-66, (B) UiO-66 12.5% HCl, (C) UiO-66 25% HCl, (D) UiO-66-NH_2_ (25%)_,_ (E) UiO-66-NH_2_ (50%), (F) UiO-66-NH_2_ (75%), (G) UiO-66-NH_2_ (100%), (H) UiO-66-NH_2_ (75%) 12.5% HCl, and (I) UiO-66-NH_2_ (75%) 25% HCl.

When considering the adsorption of the uremic toxins, the correlation of the crystal size together with their sorption capacities has to be considered. Detailed crystal sizes in a powder form and hydrodynamic diameters were determined by SEM and DLS analyses. The SEM microscopy (Figure 2) results showed that the morphology and size of prepared crystals are strongly influenced by the organic linker and modulator content used during the solvothermal synthesis. The pristine UiO-66 samples prepared with the addition of acetic acid exhibit octahedral morphology and crystal sizes in a range of 700–1600 nm. The shape of UiO-66 12.5% HCl and UiO-66 25% HCl crystals changes significantly from octahedral UiO-66, and a tendency to form agglomerated oval crystals was noticed. The formation of regular octahedral crystals was observed for −amino-functionalized UiO-66-NH_2_ crystals regardless of the content of H_2_BDC-NH_2_. The crystal shape considerably changes for UiO-66-NH_2_ as hydrochloric acid was used during the synthesis and their morphological parameters are close UiO-66 12.5% HCl and UiO-66 25% HCl samples.

Together with SEM results, the hydrodynamic particle size of prepared samples in aqueous was determined (Table 1 and Figure S10). We observed a decrease in the particle size while comparing the hydrodynamic particle sizes of the pristine UiO-66 and UiO-66 12.5% HCl and UiO-66 25% HCl samples. The UiO-66 crystal size decreases for defective UiO-66 12.5% HCl and UiO-66 25% HCl samples. The −amino-substituted UiO-66 samples revealed considerably bigger crystal sizes from approximately 800 nm for UiO-66-NH_2_ (25%) to 515 nm for UiO-66-NH_2_ (100%) showing a decreasing tendency with an increasing amount of −amino groups within the framework.

**Figure 3:**
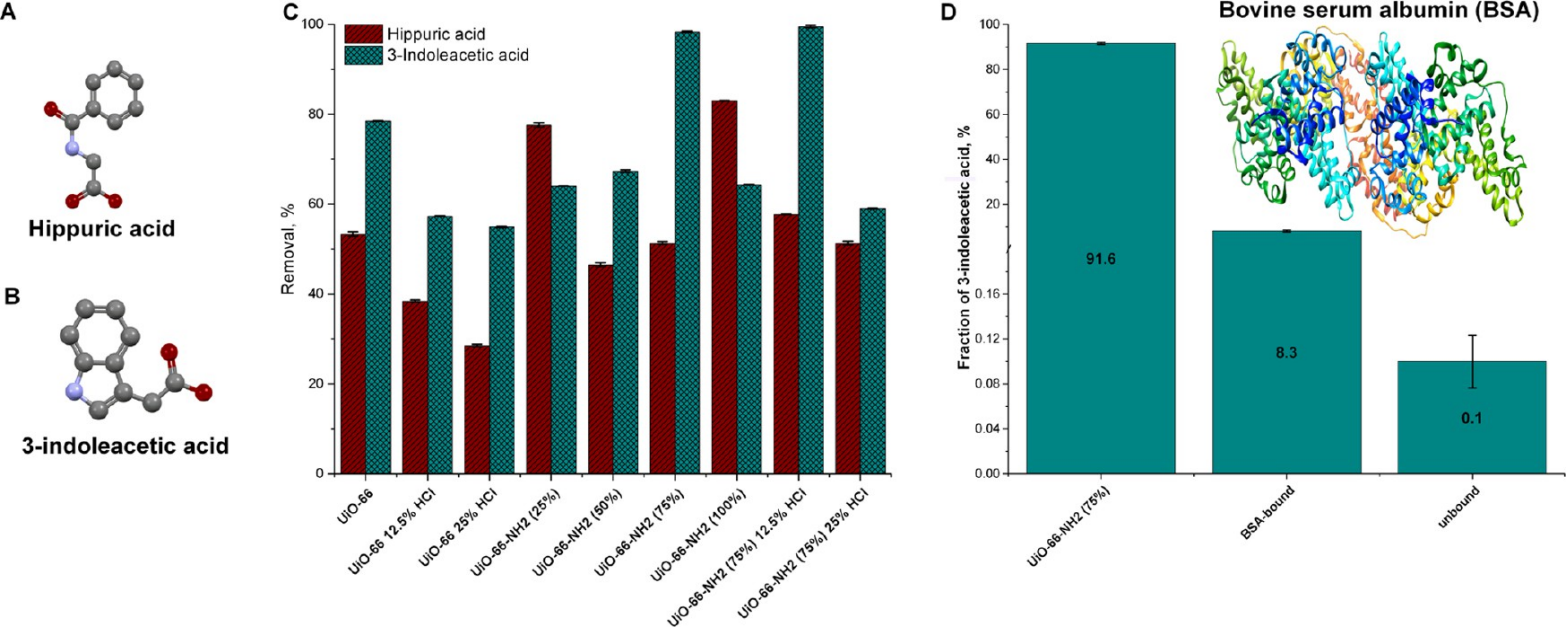
Structure of uremic toxins used in this study: (A) hippuric acid; (B) 3-indoloacetic acid; (C) removal efficiency of uremic toxins on prepared UiO-66-X samples; and (D) adsorption of 3-indoloacetic acid with UiO-66-NH_2_ (75%) and bovine serum albumin (BSA)—0.2 M NaCl aq. solution at 310 K; hydrogens are omitted for clarity.

The adsorption efficiency of two common uremic toxins (hippuric acid and 3-indoloterephthalic acid) was examined on prepared UiO-66, defective UiO-66, and UiO-66-NH_2_ samples. The adsorption efficiency results are presented in Figure 3, while detailed adsorption curves as a function of time and calculated pseudo-first-order and pseudo-second-order kinetic curves are presented in Figures S11–S16 and Tables S4 and S5. The modulated synthesis with concentrated hydrochloric acid resulted in a significant decrease in toxin adsorption. Despite an increase in structural parameter values (Table 1), application of the defect resulted in the destruction of binding sites for toxins. At the same time, functionalization of the UiO-66 material by NH_2_ resulted in ambiguous changes. Only some of the materials recorded a significant increase in toxin adsorption. The maximum adsorption capacity was achieved only for 3-indoloacetic acid for UiO-66-NH_2_ (75%) and defective UiO-66-NH_2_ (75%) 12.5% HCl. Both materials exhibited a lower SSA_BET_ value and a lower *V*_micro_ value in comparison with pristine UiO-66 (Table 1). Nevertheless, the adsorption capacity of 3-indoleacetic acid was 20–21% higher in these materials than in UiO-66. The reason for this may be that, despite a significant change in the volume of pores, their distribution has changed considerably, which resulted in an increase in diffusion resistance. The removal of 3-indoloacetic acid on the remaining MOF samples was in a 60–80% range. In the case of hippuric acid, the maximum uptake was achieved for UiO-66-NH_2_ (100%) and UiO-66-NH_2_ (25%) and was equal to 83 and 77%, respectively. The rapid adsorption of hippuric acid on −amino-functionalized UiO-66 samples can be observed in the first 10 min of adsorption (Figure S11B).

The search for efficient uremic toxin adsorbents must take into account the efficiency of the adsorption of uremic toxins to the human serum albumin (HSA). In patients with chronic kidney disease, approximately 80% of uremic toxins are bound to the HSA. To determine the adsorption efficiency of the 3-indoloacetic acid bound to amino acids, we measured the removal of the 3-indoloacetic bound to the bovine serum albumin (BSA). The BSA was used as a model system, similar to the HAS, since human serum albumin contains one tryptophan residue compared to two in BSA. The procedure of the determination of the removal of uremic toxin bound to serum albumin was adapted from the work of Kato et al.^9^ The results of the competitive adsorption of BSA-bound 3-indoloacetic acid are shown in Figure 3D. To compare the results of the competitive adsorption and the amount of BSA-bound 3-idoloacetic acid, we performed a blind trial without UiO-66-NH_2_ (75%). We determined that 97 ± 0.3% of 3-indoloacetic acid was bound to the BSA. After adding UiO-66-NH_2_ (75%) to the solution, 91.6 ± 0.48% was removed from the solution while the fractions of BSA-bound and BSA-unbound were 8.3 ± 0.48 and 0.1 ± 0.02%, respectively.

To illustrate the effect of defect engineering on the adsorption ability of two most efficient samples UiO-66-NH_2_ (75%) and UiO-66-NH_2_ (75%) 12.5% HCl, the adsorption of 3-indoloacetic acid was performed on alternated solid/liquid ratio. The results of the adsorption of 3-indoloacetic acid over 0.5 mg of UiO-66-NH_2_ (75%) and UiO-66-NH_2_ (75%) 12.5% HCl are shown in Figure S17. It may be seen that, for UiO-66-NH_2_ (75%) 12.5% HCl, rapid uremic toxin removal may be observed in the first 20 min of the adsorption process, while for UiO-66-NH_2_ (75%), the adsorption proceeds smoothly, reaching its maximum after 120 min of adsorption. The comparison between maximum adsorption of 3-indoloacetic acid on UiO-66-NH_2_ (75%) and UiO-66-NH_2_ (75%) 12.5% HCl shows that at the early stage of the adsorption process, an increased pore volume of UiO-66-NH_2_ (75%) 12.5% HCl favors rapid adsorption but results in a decrease in adsorption sites, which can be observed in a slightly decreased overall uremic toxin removal (approximately 20 μmol/g lower adsorption in comparison with UiO-66-NH_2_ (75%)).

The experimentally obtained equilibrium sorption data were also fitted into Langmuir and Freundlich adsorption isotherms (Figures S18 and S19, Table S6). The values of equilibrium adsorption were calculated from Langmuir and Freundlich equations (eqs 10 and 11, Supporting Information) and presented in terms of adsorption equilibrium constant *k*_L_ and adsorption capacity for the Langmuir model, and adsorption equilibrium constant *k*_F_ and *n* for the Freundlich model. The coefficient of determination *R*^2^ for both hippuric acid and 3-indoloacetic acid adsorption isotherms is close to 1 for the Freundlich adsorption model.

The recyclability of UiO-66-NH_2_ (75%) and UiO-66-NH_2_ (75%) 12.5% HCl in subsequent cycles of adsorption of uremic toxins was determined for 3-indoloacetic acid (Figure S20). The recyclability of the adsorption on any of the adsorbent is an important feature that is required for the MOF materials to be considered for artificial kidney application.^19^ The adsorption of 3-indoloacetic acid on UiO-66-NH_2_ (75%) and UiO-66-NH_2_ (75%) 12.5% HCl on the first cycle was close to 100% and diminished smoothly after each of the recycling cycles. After the second adsorption cycle, the adsorption efficiency decreases to approximately 80% for UiO-66-NH_2_ (75%) and remains constant after the third cycle. For the UiO-66-NH_2_ (75%) 12.5% HCl, the maximum adsorption reached after the second and third cycles was 76 and 70%, respectively.

The final step was to check the safety of the prepared UiO-66 materials; therefore, to fully confirm the low toxicological profile of UiO-66-based samples, we have applied for the first time *in vitro* experimental models for cytotoxicity toward cells potentially exposed to the direct contact with our materials, i.e., epithelial (HaCaT) and kidney (Vero, HEK-293) cell lines. HaKaT cells (keratinocyte cells) are found in the deepest basal layer of the stratified epithelium. In fact, they are the principal cell type of the epithelium and comprise about 90% of the total cell population of epithelium and even up to 95% of the cells in the epidermis.^29^ Noteworthy, HaCaT cells are the most commonly used cell lines for toxicological assessment. Vero and HEK-293 are normal kidney-derived cells, where Vero denotes kidney epithelial cells extracted from an African green monkey and HEK-293 denotes human embryonic kidney cells.

As the compounds used for hemodialysis have the greatest direct contact with blood, we decided to investigate the behavior of the tested compounds toward selected blood morphotic elements. If a compound shows toxicity to blood cells, then it cannot be used for hemodialysis purposes. Thus, the toxic effects of tested MOFs should be tested against RBCs with regard to the blood system prior to other cells it may have contact with. Hemolysis is connected with the disintegration of RBCs, and the test detects the leaking of hemoglobin, an important protein responsible for oxygen transport, into the plasma. When hemoglobin is released via hemolysis, it shows toxic effects on the vascular, myocardial, renal, and central nervous system tissues as a vasoactive and redox-active protein.^30^ Numerous studies have found good correlations between *in vitro* hemolysis tests and *in vivo* toxicity by the hemolytic effect.^31^ Thus, our study is the first investigation of the *in vitro* toxicity of UiO-66-based molecules using the outcomes of hemolysis tests to fully confirm their low toxicological profile.

**Figure 4:**
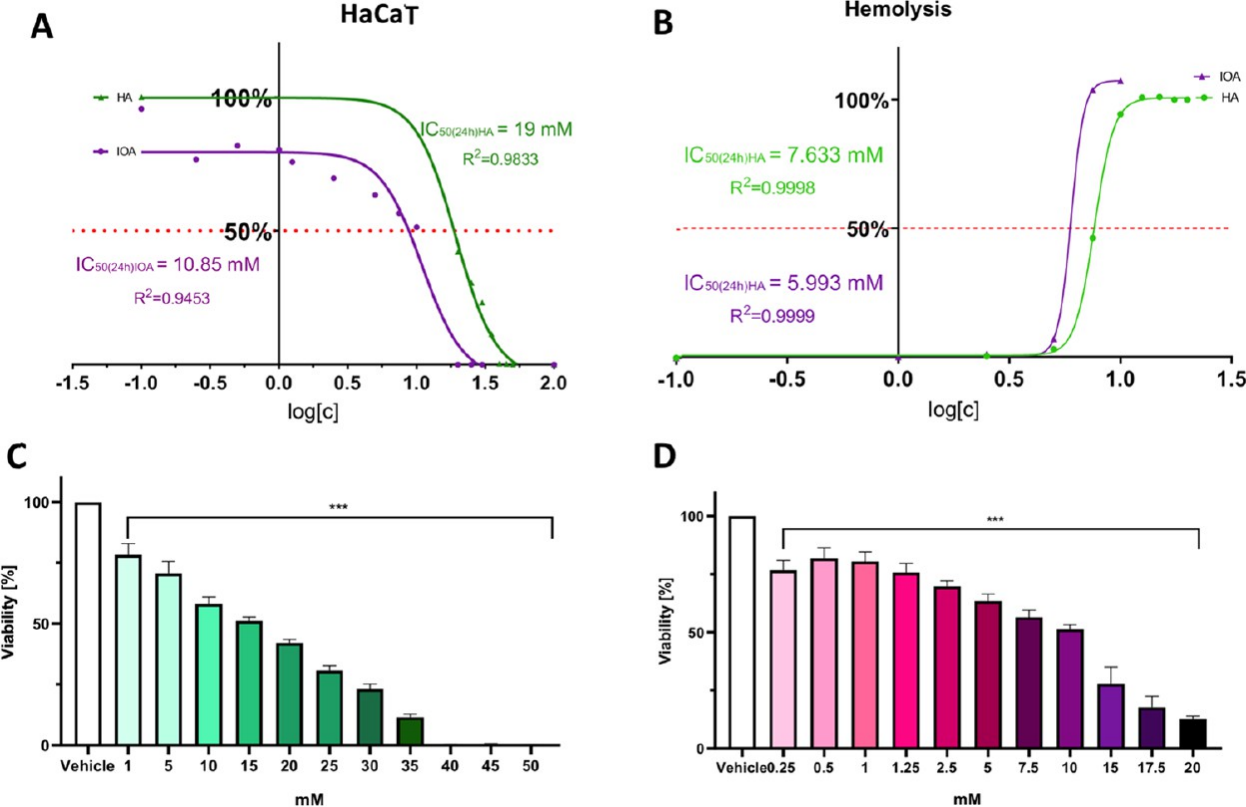
(A) Evaluation of IC_50_ for hippuric and 3-indoleacetic acids on HaCaT cell line and (B) evaluation of IC_50_ for hippuric acid and 3-indoleacetic acid in hemolytic activity test. Viability (%) results for hippuric (C) and 3-indoleacetic acids (D) on HaCaT cell line; mean values ± SD, ***p < 0.001, Tukey test.

The first step in that experiment was to evaluate IC_50_ values of exemplary uremic toxins for further studies. The evaluated values of IC_50_ were 19.71 and 7.633 mM for hippuric acid (HA), and 11.25 and 5.993 mM for 3-indoleacetic acid (IOA) for HaKaT and RBCs, respectively (Figure 4).

**Figure 5:**
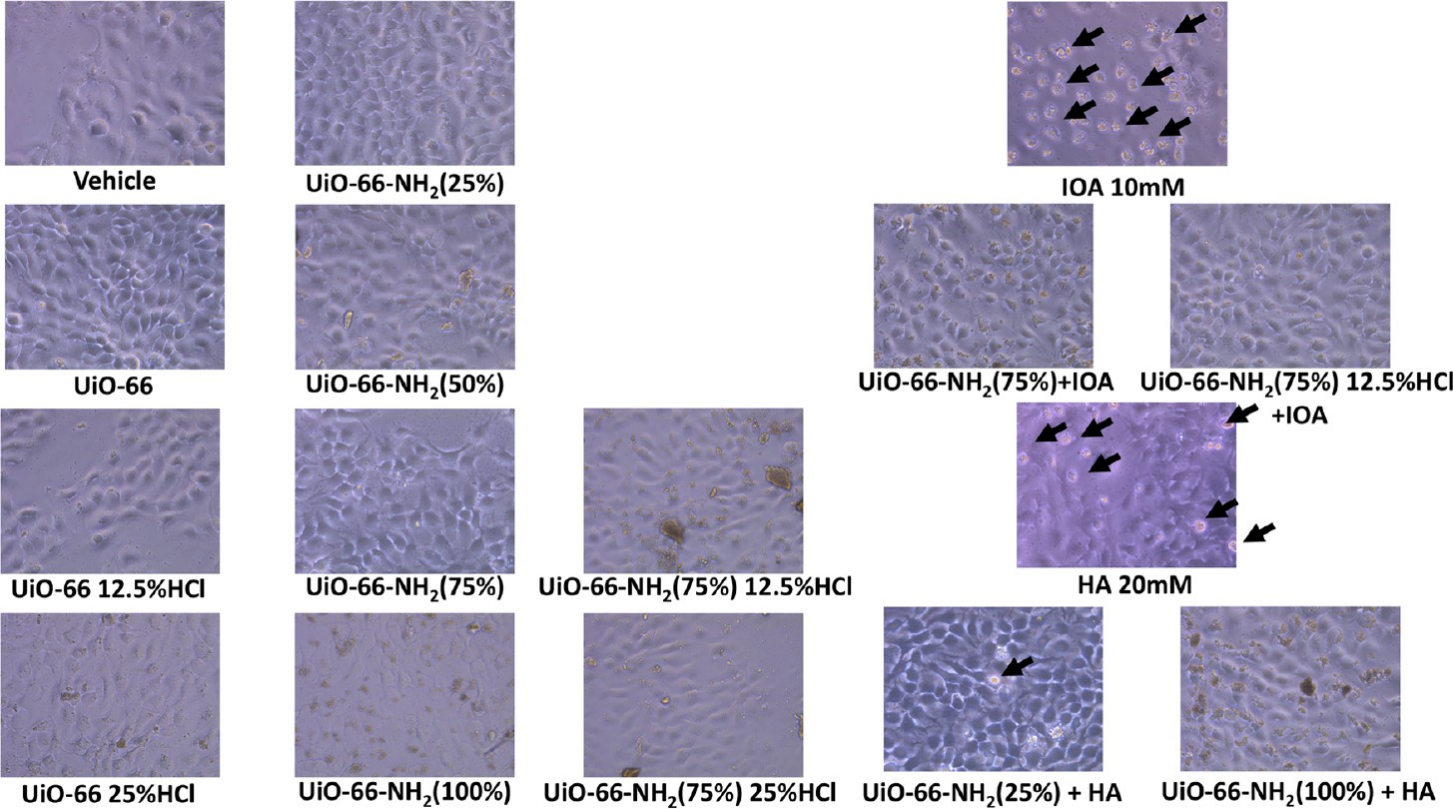
Images of HaCaT cells after 24 h treatment with UiO-66 samples without and with hippuric and 3-indoloacetic acids. Black arrows show dead cells.

**Figure 6:**
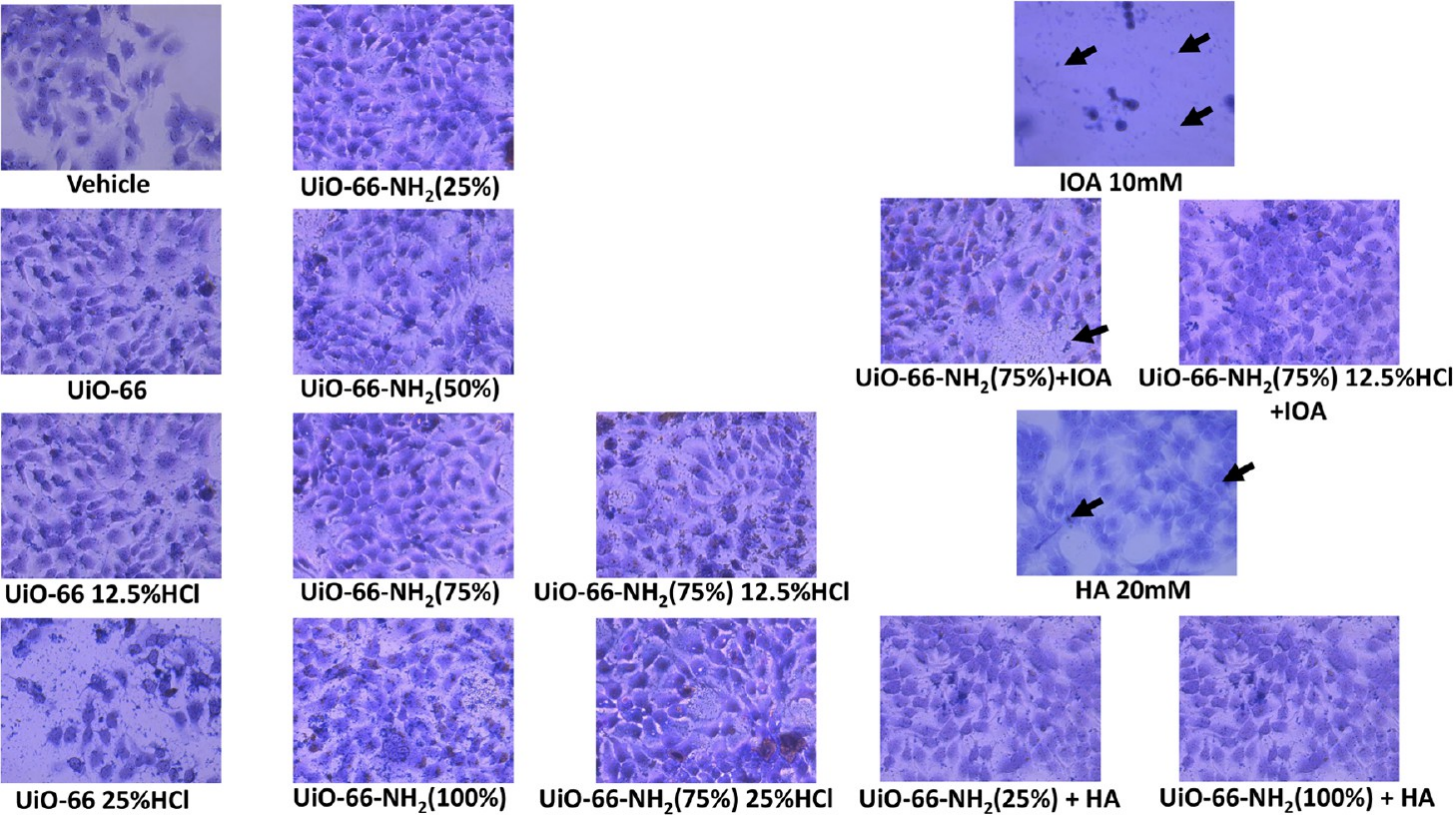
Images of HaCaT cells stained with H& E after 24 h treatment with UiO-66 samples without and with hippuric and 3-indoloacetic acids. Black arrows show dead cells.

Then, we used these concentrations of uremic toxins (approximately 20 mM for HA and 10 mM for IOA) in the experimental safety tests of UiO-66 samples versus epithelium cells. Thus, we incubated HaCaT cells with a series of UiO-66 materials at a concentration of 1 mg/mL without and with HA and IOA at their IC_50_ for 24 h, and then we observed the morphology of cells to establish cell toxicity. Images of these cells without and with H& E staining (Figures 5 and 6) show that UiO-66 samples exert no cytotoxic effect on the cells. Moreover, we have observed their cytoprotective action against the activity of uremic toxins, which proves their effectiveness and safety of application.

To prove the low cytotoxicity of examined materials versus kidney cells and kidney epithelial cells, we performed the same experiment on the HEK-293 cell line and the Vero cell line. The obtained results (cf. Figures S21–S24, Table S7), similar to that in the case of HaCaT cells, finally confirmed the low-cytotoxicity profile of the tested compounds.

**Figure 7:**
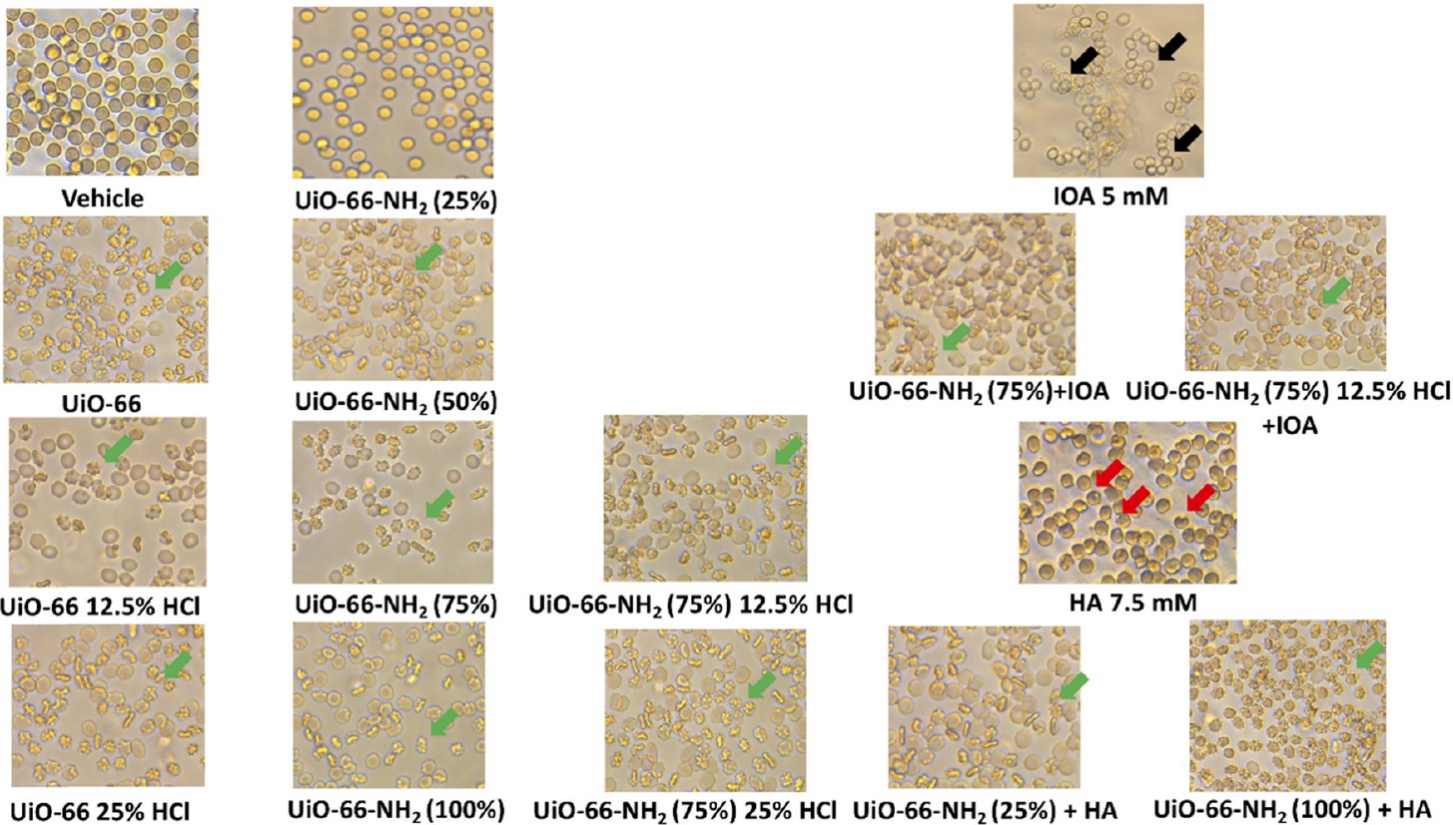
Images of the hemolytic activity test performed on human erythrocytes. Green arrows show echinocytes, red arrows show disintegrating erythrocytes, and black arrows show disintegrated erythrocytes.

The results of our study (Figure 7, Table 2) proved the high-safety profile of examined compounds versus RBCs, as the percentage of hemolysis of RBCs exposed to UiO-66 showed hematological toxicity less than 5%, while most of them did not exceed the value of 3% (cf. Table 2).

**Table 2:** Hemolytic Activity of the Prepared Samples

sample	% of hemolysis (mean ± SD)
UiO-66	3.616 ± 0.529
UiO-66 12.5% HCl	0.562 ± 0.105
UiO-66 25% HCl	4.358 ± 0.235
UiO-66-NH_2_ (25%)	3.639 ± 0.425
UiO-66-NH_2_ (50%)	4.964 ± 0.286
UiO-66-NH_2_ (75%)	2.433 ± 1.271
UiO-66-NH_2_ (100%)	2.613 ± 0.187
UiO-66-NH_2_ (75%) 12.5% HCl	2.860 ± 0.132
UiO-66-NH_2_ (75%) 25% HCl	3.616 ± 0.529
IOA 7.5 mM	91.165 ± 3.215
UiO-66-NH_2_ (75%) + IOA	4.537 ± 0.123
UiO-66-NH_2_ (75%) 12.5% HCl + IOA	3.459 ± 0.174
HA 7.5 mM	44.010 ± 2.303
UiO-66-NH_2_ (25%) + HA	2.329 ± 0.370
UiO-66-NH_2_ (100%) + HA	2.007 ± 0.125

## Discussion

The present work is a systematic study connected with the search for effective materials based on the UiO-66 structure for artificial kidney application. Our results clearly demonstrate that effective sorption of uremic toxins is a derivative of a number of factors, ranging from the type of toxin, MOF structure, method of synthesis, structural parameters, particle size, and the type and concentration of functional groups in the MOF structure.

Starting from the determination of the crystallinity of prepared samples, at first glance, no differences in diffractograms were observed. The PXRD patterns revealed subtle changes coming from several structural changes in the structure of UiO-66-modified materials. The PXRD patterns revealed broad weak reflections at 2θ ∼ 4 and 6°, indicating the presence of missing-linker/cluster nanoregions in the prepared samples (Figure S1).^32^ This phenomenon was specifically observed for samples prepared during modulated synthesis with the addition of hydrochloric acid (cf. Figure S1A) and defective −amino-functionalized samples (Figure S1C). Additionally, on PXRD for UiO-66-NH_2_ (100%), we found considerable peak broadening, which suggests the presence of smaller crystals.^26^ Indeed, taking into account the results of the hydrodynamic particle size determined by DLS and SEM analyses, the tendency of formation of smaller crystals with an increasing content of 2-aminoterephthalic acid used during modulated synthesis was confirmed. The impact of modulator concentration or the zirconium source on crystal size and morphology has been recently reported in several papers.^26,33,34^ Schaate et al.^26^ examined the influence of benzoic acid on UiO-66 and UiO-66-NH_2_ crystal size using the DLS method and XRD peak broadening. They found that, in the case of UiO-66, the crystal size increased with increasing amount of benzoic acid, which was a derivative of the formation of zirconium–benzoic acid complexes. Conversely, −amino-functionalized UiO-66-NH_2_ was nearly unaffected by the addition of the modulator (0–30 equiv), exhibiting a constant crystal size of approximately 100 nm. Comparison of our results with the results of Schaate et al.^26^ leads to the conclusion that the influence of the modulator on crystal size is not straightforward and has to be taken into account during the material development. However, the results shown here clearly exhibit that the adsorption of uremic toxins over a metal–organic framework is influenced by numerous factors optimized during the synthesis procedure. Considering multiparameter approach in the optimization of UiO-66-based materials including defect engineering and −amino functionalization and combined approach, the interaction between the uremic toxin and the adsorption center and the interaction between the uremic toxin and the MOF structure seem to have a profound effect.^9^

The analysis of the thermogravimetric profiles for pristine and −amino-functionalized UiO-66 samples showed that the MOF decomposition temperature increases with decreasing H_2_BDC-NH_2_ loading during mixed-linker synthesis, which is in good agreement with Chavan et al.^21^ Randomly distributed linkers in mixed-linker UiO-NH_2_ materials, i.e., UiO-66-NH_2_, UiO-66-NH_2_ (75%) 12.5% HCl, and UiO-66-NH_2_ (75%) 25% HCl samples, were confirmed by the comparable TGA profiles and considerably different shapes of DSC curves.^21^ However, it must be pointed out that despite the fact that both UiO-66-NH_2_ (75%) 12.5% HCl and UiO-66-NH_2_ (75%) 25% HCl samples showed similar TGA trends, both samples are evidently rich in structural defects, which is confirmed by the lower TGA curve in comparison to parent UiO-66-NH_2_ (75%) (cf. Figure S5).^21^

Complementary information on the structure of the received materials is provided by the results of dissolution/^1^H NMR. The dissolution/^1^H NMR method proposed by Shearer et al.^28^ provides information about the impurities that may be incorporated into the MOF framework during the modulated synthesis. According to Shearer et al.,^28^ the modulated samples may be contaminated with a considerable amount of monocarboxylic modulator or from DMF used as a solvent, which hydrolyzed to formate and dimethylamine in a highly basic 1 M NaOH medium. Despite the fact that our samples were activated at 200 °C, which successfully removed both formates and dimethylamine from UiO-66 pores, acetates and formates are still present in small amounts in −amino-functionalized samples. The reason for that is unknown; however, we can speculate that there is a strong interaction between formates and −amino groups inside the UiO-66-NH_2_ framework. A comparison of the modulator/BDC, formate/BDC, and total modulator/BDC ratio (Table S3) confirms the presence of acetates and formates in the MOF framework. The comparison of total modulator/BDC ratios found in the work by Shearer et al.^28^ and the results in this study is justified only for UiO-66 prepared with the addition of 9.2 mL of acetic acid and the sample denoted by Shearer et al.^28^ as UiO-66 36 Ac. synthesized by the addition of 7.626 mL of acetic acid during the modulated synthesis. The modulator/BDC ratio in both samples was at a similar level except the acetate/BDC ratio, which is twice higher in the case of our sample. The reason for that is the derivative of 2 mL more volume of acetic acid used in the case of UiO-66. The acetate/BDC ratio oscillates around 0.19, with the exception of UiO-66-NH_2_ (100%), where it reaches the value of 0.33. The increased content of acetates in the framework is most likely caused by the reduced average pore diameter determined, which makes it difficult to wash out and activate the sample.

The results of the adsorption studies in conjunction with the analyses of their structure indicate that, in particular, functionalization with amino groups can have a positive effect on toxin uptake. Similar trends were observed in the case of 3-indoleacetic acid. The defect provided in this case binding sites for toxins and higher adsorption efficiency. However, the correlation of the values of the parameters describing the pore space of the materials (SSA_BET_, *V*_micro_, and *D*) here is not clearly related to their abilities in toxin adsorption (Figure S25A–C). Despite the possibility of optimizing the pore size in the MOF structure during modulated synthesis, increasing the pore volume considerably decreases the number of binding sites for the adsorption of uremic toxins. The reduced number of available adsorption centers and the presence of acetates and formates compensating structural defects result in a significant reduction in sorption capacity.

Additionally, as was previously described in the work by Kato et al.,^9^ the great sorption capacity of *p*-cresyl sulfate over NU-1000 is caused by electrostatic and π–π interactions between uremic toxin and pyrene linker in NU-1000. As demonstrated, the *p*-cresyl sulfate adsorption on the NU-1000 occurred on hydrophobic adsorption sites located close to Zr_6_ hydroxyl groups, which were able to bond sulfate groups of the adsorbate by hydrogen bonding. In this study, as well as π–π interactions between adsorbed uremic toxins and H_2_BDC in the case of UiO-66, we also observed maximization of the adsorption efficiency in the −amino functionalization of the UiO-66 structure and the synergic effect of amino functionalization and defect generation. The former is clearly visible for the adsorption of 3-indoleacetic acid over UiO-66-NH_2_ (75%) 12.5% HCl, where both −amino functionalization and defect generation were optimized. Moreover, the adsorption of hippuric acid over UiO-66-NH_2_ (25%) and UiO-66-NH_2_ (100%) demonstrates that the adsorption capacity is influenced by the number of −amino groups substituted to the UiO-66-NH_2_ structure. Two extreme values of the −amino groups (25 and 100%) in functionalized UiO-66-NH_2_ show that the maximum adsorption values in the case of hippuric acid are achieved by complete substitution of H_2_BDC by H_2_BDC-NH_2_. However, the adsorption of 3-indoleacetic acid indicates that this molecule requires the adsorbed structure to be optimized in both −amino substitution and defect generation by modulated synthesis with HCl. The comparison of modeled electron densities for “defect-free” (Figure S26) and missing-linker (Figure S27), missing node (Figure S28), and missing-linker and node UiO-66 (Figure S29) would at first glance suggest the profound effect on defect generation of adsorption efficiency. The generated defect associated with linker/node removal through modulated synthesis generated void spaces, which increases the overall adsorption capacity^18^ through the generation of wider channels in the UiO-66 structure (cf. pore size distribution, Figure S4 insets). Indeed, the complete understanding of the mechanisms of the uremic toxins over modified UiO-66 samples would require in-depth DFT studies on the adsorption sites and the location of guest molecules in prepared materials.^9,35^

In considering the potential application of MOF materials for artificial kidney purposes, the uremic toxin adsorption ability should be considered on composite materials through which the blood will be passing during the hemodialysis. The recent work by Abdelhameed et al.^19^ describes the synthesis route of the preparation of UiO-66-(COOH)_2_ that was grown directly on cotton fabric. The resulting UiO-66-(COOH)_2_@cotton fabric composite was tested in the adsorption of creatinine. The high adsorption efficiencies obtained by Abdelhameed et al.^19^ cannot be compared with the results from this study due to the differences between creatinine, hippuric acid, and 3-indoloacetic acid. However, in our opinion, the results from the work of Kato et al.,^9^ Abdelhameed et al.,^19^ and our own work suggest that achieving high sorption effects of uremic toxins will require the use of a composite material consisting of a series of MOFs selectively adsorbing a given family of uremic toxins. Therefore, in our opinion, a potential solution should have high sorption parameters, low cytotoxicity, and hemotoxicity as well as the ease of MOF material synthesis and low material cost.

Apart from optimizing the structure of MOF through its modulated synthesis and introducing functional groups, an important parameter is its cytotoxicity and hemotoxicity. Both of these factors are limiting in the search for new materials for artificial kidney applications. Since the results of cytotoxicity are quite obvious, the hemolytic activity of prepared samples should be discussed. In blood samples incubated with UiO-66 and related materials, a change can be observed in the shape of the RBC membrane characterized by numerous small, evenly spaced spikes (they look like sea urchins). Such a change of erythrocytes shape is called echinocytosis and is a reversible condition of RBCs, often caused by the presence of anticoagulant (mainly EDTA) or, as in our case, developed during hemolysis. The number of echinocytes is related to the increase in viscosity of blood that occurs during hemolysis. Echinocytes return to their normal RBC shape as the hemolytic factor is removed from direct contact with blood. This condition, together with cytotoxicity results, is the final confirmation of the low toxicological profile of the presented UiO-66 samples.

## Conclusions

The influence of mixed-linker synthesis of UiO-66 to obtain UiO-66-NH_2_ materials varying with the final content of −amino groups together with modulated synthesis with HCl on adsorption efficiency of uremic toxins was investigated in this study. The optimization of synthesis parameters such as the H_2_BDC/H_2_BDC-NH_2_ ratio and the amount of concentrated HCl at the preparation step allowed crystalline UiO-66-NH_2_ materials with high uremic toxin sorption capacities to be obtained. The maximum sorption capacity for hippuric acid was achieved for UiO-66-NH_2_ with 25 and 75 mol % −amino groups substituted in the UiO-66 structure and channels within the 5–9 and 14–18 Å ranges. The maximum adsorption capacity for 3-indoloacetic acid was achieved for UiO-66-NH_2_ (75%) HCl and modulated UiO-66-NH_2_ (75%) 12.5% HCl samples with pores in the ranges of 5–8 and 17–22 Å, respectively. Compared with the Zr MOF materials with the same topology, the obtained UiO-66 and modified UiO-66-NH_2_ samples reveal analogous sorption capacity to NU-1000. The adsorption isotherms for hippuric acid and 3-idoloacetic acid were fitted to the Langmuir and Freundlich models to obtain kinetic parameters. An almost linear correlation of both uremic toxins to the Freundlich model was found.

The uremic toxin adsorption results for modified UiO-66 and UiO-66-NH_2_ materials prepared by mixed-linker modulated synthesis show that appropriate modification of the synthesis step to obtain defective amino-functionalized UiO-66 materials is a key step in the preparation of efficient sorbents for uremic toxins. The cytotoxicity tests performed over HaCaT, Vero, HEK-293, and RBCs cells showed that the prepared UiO-66 samples revealed no cytotoxic effect. Furthermore, their cytoprotective effect against hippuric and 3-indoloacetic acid as a model uremic toxin has proven that they can be considered as potentially safe for hemodialytic purposes.

In conclusion, for the first time, we have described a complementary approach for the synthesis, characterization, and *in vitro* toxicological evaluation of UiO-66-based materials for dialysis purposes. And the outcomes positively anticipate further study in this field.

## Supporting Information Available

Experimental details; characterization details; PXRD patterns for prepared UiO-66 and UiO-66-NH_2_ samples; UV–Vis absorbance spectra of UiO-66-NH_2_ (*X*%) and UiO-66-NH_2_ (*X*%) *Y*% HCl samples diluted in 1 M aq. NaOH used for the determination of H_2_BDC-NH_2_; low-temperature N_2_ sorption isotherms; TGA/DSC results; *in situ* DRIFT; dissolution ^1^H NMR spectra; DR UV–Vis spectra of prepared samples; hydrodynamic particle size distribution; adsorption efficiency of 1 mg of prepared UiO-66 samples in 1.5 mL of 0.1 mM hippuric acid as a function of time; pseudo-first-order kinetic model of adsorption of hippuric acid on the prepared UiO-66 samples; pseudo-second-order kinetic model of adsorption of hippuric acid on the prepared UiO-66 samples; adsorption efficiency of 1 mg of prepared UiO-66 samples in 1.5 mL of 0.1 mM 3-indoloacetic acid as a function of time; pseudo-first-order kinetic model of adsorption of 3-indoloacetic acid on the prepared UiO-66 samples; pseudo-second-order kinetic model of adsorption of 3-indoloacetic acid on the prepared UiO-66 samples; Langmuir and Freundlich plots for hippuric acid and 3-indoloacetic acid for selected samples; synthesis details of mixed-linker defective UiO-66 samples; UV–Vis quantitative analysis results of H_2_BDC-NH_2_; kinetic parameters of pseudo-first-order and pseudo-second-order models for hippuric acid; kinetic parameters of pseudo-first-order and pseudo-second-order models for 3-indoloacetic acid; Langmuir and Freundlich parameters for the adsorption of uremic toxins on the prepared MOF samples; 3-indoloacetic acid adsorption recyclability; images of Vero cells stained with H& E; images of HEK-293 cells stained with H& E; correlations between toxin removal efficiencies with structural parameters; structural model of “defect-free” UiO-66; structural model of UiO-66 missing-linker regions; structural model of UiO-66 missing node regions; and structural model of UiO-66 missing-linker and missing node regions (PDF)
