# Supplementary material for: In Search of Effective UiO-66 Metal–Organic Frameworks for Artificial Kidney Application
Source: ACS Appl Mater Interfaces. 2021 Sep 14;13(38):45149–60. doi: 10.1021/acsami.1c05972 (PMC8485328; doi:10.1021/acsami.1c05972)
Supplement: Supplementary file 1 — am1c05972_si_001.pdf [file am1c05972_si_001.pdf]

Supporting Information

In search of effective UiO-66 metal organic frameworks for artificial kidney application

*Klaudia Dymek [a], Grzegorz Kurowski [a], Łukasz Kuterasiński [b], Roman Jędrzejczyk [c], Magdalena Szumera [d], Maciej Sitarz [d], Anna Pajdak [e], Łukasz Kurach [f], Anna Boguszewska-Czubara [g], Przemysław J. Jodłowski [a]**

[a] Klaudia Dymek, Grzegorz Kurowski, Przemysław J. Jodłowski*, Faculty of Chemical Engineering and Technology, Cracow University of Technology, Warszawska 24, 30-155 Kraków, Poland

[b] Łukasz Kuterasiński, Jerzy Haber Institute of Catalysis and Surface Chemistry, Polish Academy of Sciences, Niezapominajek 8, 30-239 Kraków, Poland

[c] Roman Jędrzejczyk, Małopolska Centre of Biotechnology, Jagiellonian University, ul. Gronostajowa 7A, 30-387 Kraków, Poland

[d] Magdalena Szumera, Maciej Sitarz, Faculty of Materials Science and Ceramics, AGH University of Science and Technology, Mickiewicza 30, 30-059 Kraków, Poland

[e] Anna Pajdak, Strata Mechanics Research Institute, Polish Academy of Sciences, Reymonta 27, 30-059 Kraków, Poland

[f] Łukasz Kurach, Independent Laboratory of Behavioral Studies, Medical University of Lublin, 4A Chodzki Str, Lublin, 20-093, Poland

[g] Anna Boguszewska-Czubara, Department of Medical Chemistry, Medical University of Lublin, 4A Chodzki Str, Lublin, 20-093, Poland

Corresponding Author

* Przemysław J. Jodłowski, Faculty of Chemical Engineering and Technology, Cracow University of Technology, Warszawska 24, 30-155 Kraków, Poland, E-mail: pjodlowski@pk.edu.pl

Experimental section

Materials: zirconium (IV) chloride (Sigma Aldrich), terephthalic acid (H_2BDC, Sigma Aldrich, 98%), 2-amino terephthalic acid ($H_2BDC-NH_2$, Sigma Aldrich), hydrochloric acid (POOCH Poland), N,N-dimethylformamide (DMF, Chempur Poland), methanol (POOCH Poland), hippuric acid (Sigma Aldrich), 3-indoloacetic acid (Sigma Aldrich). All chemicals were reagent grade and used without purification. The deionized water was purified by ensuring water conductivity not greater than 0.05 mS.

MOF synthesis

Synthesis of pristine UiO-66 and defective UiO-66

The series of UiO-66 metal-organic frameworks was synthesized according to the procedures described elsewhere [1,2] with some modifications. In brief, the pristine UiO-66 sample was prepared by dissolving 0.19 g (0.082 mmol) $ZrCl_4$ and 0.13 g (0.78 mmol) benzene-1,4-dicarboxylic acid (H_2BDC) in 81.7 ml of DMF. The resulting mixture was then ultrasonicated for 5 minutes and 9.2 ml of acetic acid was added to the solution. The mixture was then transferred to Teflon liners and kept at 120 °C for 24h. The resulting UiO-66 crystals were

separated from the solution by centrifugation. The white crystals were washed 3 times in DMF to remove unreacted precursors, and then washed 3 times with methanol to exchange the solvent. The parent sample was denoted as UiO-66.

The UiO-66 samples prepared by modulated synthesis with concentrated HCl were synthesized according to the procedure described elsewhere. [3] Briefly, 0.19 g of $ZrCl_4$ was dissolved in 18.75 ml or 12.5 ml DMF and an appropriate volme of concentrated HCl (6.25 or 12.5 ml). The resulting mixture was added to the mixture containing 0.26 g of H_2BDC dissolved in 25 ml DMF. It was then transferred to Teflon liners and kept at 120 °C for 24 h. The crystals were collected and washed according to the procedure described for pristine UiO-66. The defective UiO-66 samples were marked with the suffix Y% HCl, corresponding to HCl vol% used during the modulated synthesis.

Synthesis of parent UiO-66-NH_2 and defective UiO-66-NH_2

The series of UiO-66-NH_2 and defective UiO-66-NH_2 were synthesized according to the procedure described for pristine UiO-66, with the difference that H_2BDC-NH_2 was mixed with H_2BDC in the appropriate ratios to give the final values of H_2BDC-NH_2 equal to 25, 50, 75 and 100 wt%. The samples containing -NH_2 groups were marked UiO-66 with a suffix (X%), corresponding to wt% of the H_2BDC-NH_2 organic linker used during the synthesis.

The mixed amine functionalized defective UiO-66 samples were prepared for UiO-66 (75%) by adding the appropriate volme of concentrated HCl (6.25 or 12.5 ml) to the sythesis reagents. The resulting samples were marked as UiO-66-X-Y where X=75% NH_2 and Y= 12.5 or 25 vol% HCl, respectivelly. The synthesis details for all prepared samples are summarized in Table S1.

Characterization

The crystallinity of prepared UiO-66 samples was determined by powder x-ray diffractometry (PXRD) using an X'Pert Pro MPD (PANalytical) diffractometer with CuKα radiation. The

theoretical PXRD patterns for pristine UiO-66 and UiO-66-NH_2 were calculated using Mercury 2020 3.0 software based on .cif files for UiO-66 [4] and UiO-66-NH_2 [5]. The .cif files for UiO-66 missing linker and UiO-66 missing node were prepared on the basis of [4] and modified in VESTA 3.4.4 software. [6] The corresponding MOF structures were visualized by using Mercury 2020 3.0 and VESTA 3.4.4 software. [6] The structures of UiO-66 for idealized UiO-66 crystal and defective samples showing the missing linker and missing node were presented using OLEX2 software[7] (Figures S20-S22A). The electron density maps were simulated from modified .cif files using VESTA 3.4.4 software [6] (Figures S20-S22 B).

The MOFs samples were subjected to structural analyses by the low-pressure nitrogen adsorption (LPNA) method using an ASAP 2020 volumetric analyzer operating under isothermal conditions. The measurement took place at 77 K, in the absolute pressure range of 0-0.1 MPa and relative pressure range of $0< p/p_0 <0.996$. Samples were prepared for analysis by degassing for 12 hours under a vacuum at 423 K. The specific surface area was determined using the BET (SSA_{BET}) and Langmuir (SSA_L) methods, as well as the total pore volume and pore size distribution using the NLDFT method for cylindrical pores. The influence of additives on the size of the multilayer (BET) and monolayer (Langmuir) specific surface area and on the pore space structure of the prepared MOFs was determined through structural studies.

The molecular structure of prepared UiO-66 samples was examined by means of *in situ* Diffuse Reflectance Fourier Transform Infrared Spectroscopy (*in situ* DRIFT) using a Thermo is10 spectrometer equipped with MCT detector. Prior to analysis, MOF samples (approx. 20 mg) were placed in high temperature DRFIT reactor (Praying Mantis High Temperature Reaction Chamber) and activated under 50 ml/min Ar flow at 110 °C for 1h. The spectra were collected in the 4000-650 cm^{-1} range under dehydrated conditions at 110 °C, under a 50 ml/min Ar flow by averaging 128 scans with 4 cm^{-1} resolution. The experimental concentration of H_2BDC-NH_2 in -amino functionalized UiO-66-NH_2 samples was determined by means of UV-Vis

spectroscopy, using the method previously reported elsewhere, [8] with some modifications. In brief, approx. 20 mg of -amino functionalized UiO-66 sample was digested in 35 ml of 1M NaOH solution and subsequently ultrasonicated in an ultrasonic bath (Ulsonix PROCLEAN 2.0M) for 30 min to allow complete MOF digestion. The MOF mass (actual MOF mass) used for calculation of mol% of H_2BDC-NH_2 in -amino functionalized UiO-66-NH_2 samples was assumed to be 70% of the weighed MOF sample and represent MOF weight excluding solvent. The theoretical mass of H_2BDC-NH_2 was calculated according to the formula: [8]

$$Theoretical\ mass\ of\ H_2\mathrm{BDC}-NH_2 = \frac{6X \cdot M.W._{H_2\mathrm{BDC}-NH_2}}{Thoretical\ M.W.of\ MOF} x\ actual\ mass\ of\ MOF \quad (1)$$

where X corresponds to wt% of H_2BDC-NH_2 used during synthesis and is equal to 0.25, 0.5, 0.75 and 1 for UiO-66-NH_2 (25%), UiO-66-NH_2 (50%), UiO-66-NH_2 (75%), UiO-66-NH_2 (100%), respectively. The theoretical molecular weight (M.W.) was calculated assuming "ideal" $Zr_6(O)_4(OH)_4(BDC\text{-}NH_2)_{6X}(BDC)_{6-6X}$. Theoretical concentration of H_2BDC-NH_2 digested in 35 ml of 1m NaOH was calculated according to the formula: [8]

$$Theoretical\ conc.of\ H_2\mathrm{BDC}-NH_2 = \frac{Theoretical\ mass\ of\ H_2\mathrm{BDC}-NH_2}{M.W.of\ H_2\mathrm{BDC}-NH_2 \cdot 35 \cdot 10^{-3} L} \quad (2)$$

The experimental mol% of H_2BDC-NH_2 in prepared -amino functionalized samples was calculated according to the formula: [8]

$$mol\%\ H_2\mathrm{BDC}-NH_2 = \frac{Experimental\ conc.of\ H_2\mathrm{BDC}-NH_2}{Theoretical\ conc.of\ H_2\mathrm{BDC}-NH_2} \cdot X \cdot 100\% \quad (3)$$

The UV-Vis spectra of digested samples were recorded by using an AvaSpec- ULS3648 high-resolution spectrometer equipped with AvaLight-DHc deuterium-halogen light source and

Sarspec CH-MP cuvette holder for transmission UV-Vis experiments. The spectra were recorded in the 200-700 nm range. The H_2BDC-NH_2 concentration was determined by measuring the maximum absorbance at 329 nm. Prior to the UV-Vis measurements, the resulting solutes were filtered by HPPTFE-33mm-0.22µm syringe filters.

The scanning electron microscopy (SEM) of prepared MOF samples was performed by using a Nova Nano SEM 300 FEI. Prior to analysis, the MOF samples were deposited on graphite holders from ethanolic solutions.

The hydrodynamic particle size diameter of prepared UiO-66 samples was determined by using Dynamic Light Scattering Malvern Zetasizer Nano ZS equipped with 633 nm HeNe laser.

Thermal characteristics of the obtained samples were determined by DSC-TGA measurements conducted on a Netzsch STA 449 F3® operating in the heat flux DSC mode. The temperature and heat calibrations of the instrument were performed using the melting temperatures and melting enthalpies of high-purity materials (Al, Zn, Sn, Au, Ag). The samples (~6 mg) were heated in platinum crucibles at 5 ºC min^{-1} in a dry air atmosphere up to 900 ºC. Characteristic thermal parameters of the samples were determined by applying the Netzsch Proteus Thermal Analysis Program (version 5.0.0.).The TGA/DSC results were normalized with the assumption that, under experimental conditions, the corresponding residue is pure ZrO_2[9].

The molar ratio between modulator and BDC linker was determined as described elsewhere [9] by using dissolution/^{1}H NMR technique. Prior to the analysis, 20 mg of MOF was digested in 700 µl 1 M NaOD in D_2O and incubated for 24 hours. The dissolution/liquid ^{1}H NMR spectra were performed by using Bruker Avance III HD 400 MHz by averaging 8 scans and relaxation delay set to 15 seconds. The detailed description of the dissolution/liquid ^{1}H NMR principles as well as procedure for determining acetate to BDC, formate to BDC, and total modulator to molar ratios is described elsewhere [9].

Cytotoxicity

The cell proliferation assay - MTT assay

HaCaT, normal human keratinocyte cell line (RRID: CVCL_3653), VERO, monkey kidney epithelial cells (ATCC: CCL-81) and HEK-293, normal human embryonic kidney cells (ATCC: CRL-157) were cultured using DMEM with 4.5 g/L glucose and GlutaMAX^{TM} supplemented with 10% heat-inactivated FBS (v/v), 1% NEAA (v/v), sodium pyruvate (1 mM), 100 U/ml penicillin, and 100 µg streptomycin. All products were from Corning (Manassas, VA, USA). Cells were maintained at 37 °C in a humidified atmosphere of 95% air/5 % CO_2. Cultures were subcultivated every 3-4 days by trypsinization (0.25 % trypsin/EDTA). For toxicity evaluation cells were plated in complete medium (10% FBS) at density 2-4 $\cdot 10^5$ cells/ml in 96 well plates 24 h before treatment, and then exposed to the uremic toxins (hippuric acid (HA) and 3-indoloacetic acid (IOA)) in serum free medium in sextuplicate. The cells were incubated for another 24 h, and 10 µL of a 0.5 mg/mL MTT (3-(4,5-Dimethyl-2-thiazolyl)-2,5-diphenyl-2H-tetrazolium bromide) solution was added to each well, followed by incubation for 3 h at 37 °C. The supernatants were removed and 100 µl of DMSO (Sigma Aldrich) per well was added to dissolve precipitated formazan. The plate was agitated for 10 min and absorbance was measured at 560 and 620 nm using an BioTek Epoch plate reader (BioTek Instruments, United States). From cells viability values IC50 was calculated for each uremic toxin (Figure 8, Figure S17).

Visual assessment of cell morphology

To evaluate the cytotoxicity of UiO-66 samples, cell morphology was observed. As such, HaCaT cells were plated in 24-well plates at a density of 4 $\cdot 10^5$ cells/ml in a complete medium (10% FBS) 24 h before treatment, and then they were exposed to UiO-66 samples at a concentration of 1 mg/ml with and without uremic toxins: HA and IOA at their IC_{50} concentrations in a serum-free medium. A Leica MC 120 HD microscope (Heerbrugg,

Switzerland) with 40x magnification and Leica Application Suite 3.4.0 were used to monitor cell morphological changes. Images were captured after 24 h of treatment (Figure 5, Figure 6, Figure S23, Figure S24). Then, the cells were stained with haematoxylin and eosin (H&E) for better visualisation (Figure 7).

Haemolytic activity assay

The sample of human blood (provided voluntarily by the author of the manuscript) was collected in citrate (anticoagulant) containing tubes. The blood sample was centrifuged at 500 g for 10 min at 4 °C to separate erythrocytes from plasma. The supernatant was discarded and the erythrocytes were resuspended in PBS buffer and centrifuged as previously for several times until a transparent supernatant was obtained. Then the erythrocytes were suspended in PBS buffer at a final concentration of 2%. The examined compounds were added to 1 mL of the 2% erythrocyte suspension and incubated for 1 h at 37 °C. At that time the photo of the erythrocytes were taken with Leica microscope at 40x magnification. Then the samples were centrifuged at 5000g for 10 min and absorbance at a wavelength of 415 nm was measured. The percentage of haemolysis was calculated according to the following formula:

$$\text{Haemolysis (\%)} = \frac{Absorbance\ of\ sample - absorbance\ of\ blank}{Absorbance\ of\ positive\ control - absorbance\ of\ blanc} \cdot 100\% \quad (4)$$

Statistical analysis

Statistical analysis was performed using GraphPad Prism 8 software. Non-linear regression (curve fit) was used to establish IC_{50} values of HA and IOA after 24 h of incubation. The results of cell viability were expressed as a mean value as well as a standard deviation and the statistical significance of differences between the control group and the other groups was evaluated using one-way analysis of variance (ANOVA) followed by Tukey's post hoc test. Values with $p < 0.05$ were considered significant.

Sorption studies

To determine the sorption efficiency of uremic toxins as a function of time, kinetic studies of hippuric acid and 3-indoloacetic adsorption on prepared UiO-66 samples were performed by exposing 1 mg of prepared UiO-66 samples to 1.5 ml of 0.1 mM solutions of hippuric acid or 3-indoloacetic acid under thermostatic conditions (20 °C). The isotherms were determined by collecting 1.5 ml of the uremic toxins at specified time intervals. Prior to analysis, the uremic toxins solutions were filtered with 0.22 µm HP-PTFE syringe filters. The uremic toxins were analyzed by means of high-performance liquid chromatography (HPLC) for hippuric acid and by UV-Vis spectroscopy for 3-indoloacetic acid. The HPLC measurements were performed on an HPLC system equipped with a Waters 717 Plus Autosampler, a Beckman binary pump, a column thermostat and a Knauer variable wavelength monitor (UV-Vis) and Kinetex C18 HPLC column (5 µm particle, 100A, 150 × 4.6 mm^2 I.D.). HPLC was performed in reverse phase by using isocratic elution with the mobile phase of 5 mM NaH_2PO_4 and acetonitrile (90: 10 v/v %). The flow rate was adjusted at 1 ml/min, and a detection wavelength of 250 nm was used for hippuric acid. The volume of the sample collected for the chromatographic system was 20 µl. To determine the concentration of 3-indoloacetic acid, an AvaSpec-ULS3648 High-resolution spectrometer equipped with Mikropack DH-2000-BAL Deuterium-Tungsten Halogen Light Source and Sarspec CH-MP multipurpose cuvette holder was used. The 3-indoloacetic acid concentration was determined by measuring the intensity of the band at 278 nm.

The adsorption experiments performed in this study were described by using pseudo-first order and pseudo-second order models previously described elsewhere [10,11]. The parent and equilibrium amount of adsorbed uremic toxins was calculated by using the following formula:

$$q_e = (C_0 - C_e)\frac{V}{W} \quad (5)$$

where: C_o – initial, C_e – equilibrium concentrations of supernatant, V- volume of solution used (L), W- weight of the MOF sample (g).

The pseudo-first-order and pseudo-second-order models were used to calculate the adsorption kinetics. The pseudo-first-order model is described by the following equation [11]:

$$log_{10}(q_e - q_t) = log_{10}q_e - \frac{k_1 t}{2.303} \quad (6)$$

Where, q_e and q_t are the equilibrium amounts of the uremic toxins adsorbed at the time t, k_1 is the pseudo-first order rate constant (mol^{-1}).

The model for the pseudo-second-order adsorption kinetics is represented by the equation:

$$\frac{t}{q_t} = \frac{1}{k_2 q_e^2} + \frac{t}{q_e} \quad (7)$$

Where, q_e and q_t are the equilibrium amounts of the uremic toxins adsorbed at the time t, k_1 is the pseudo-second order rate constant ($g \cdot mmol^{-1} \cdot min^{-1}$). The pseudo-second order rate constant as well as equilibrium amount of the uremic toxins adsorbed at the time 't' can be calculated the linear regression by using following equations, respectively:

$$k_2 = \frac{(slope)^2}{intercept} \quad (8)$$

$$q_e = \frac{1}{slope} \quad (9)$$

The adsorption isotherms for the selected UiO-66 samples were described by commonly used Langmuir and Freundlich isotherms isotherm models. The Langmuir and Freundlich models are represented by equations 10 and 11, respectively:

$$\frac{C_e}{q_e} = \frac{1}{k_L q_{MAX}} + \frac{C_e}{q_{MAX}} \quad (10)$$

$$log q_e = log k_F + n log C_e \quad (11)$$

where: k_L - the Langmuir constant ($L \cdot \mu mol^{-1}$), k_F - Freundlich adsorption constant ($\mu mol^{1-n} \cdot L^n \cdot g^{-1}$), q_{MAX} - maximum adsorption capacity of the adsorbent ($\mu mol \cdot g^{-1}$).

The removal of 3-idoloaceticacid from bovine serum albumin solution (BSA, Sigma-Aldrich, CAS: 9048-46-8, ~66 kDa, purity ≥98%) was measured according to the procedure described in Kato *et al.* [10] with some modifications. In brief, 150 mg BSA in 6 ml of 0.2 M NaCl containing 105 µg 3-idoloacetic acid was placed in the oven for 24 h at 310 K to ensure uremic toxin bound to BSA. After 24 h, 0.66 mg UiO-66-NH_2 (75%) was added to the 1 ml solution and placed in the oven for 24 h at 310 K. Subsequently, the resulting solutions were divided into two and one half was filtered by centrifugal ultrafiltration device (Vivaspin 20 Centrifugal Ultra Filter, 10 MWCO, PES Membrane) at 5000 RCF for 10 min. The other half was heated at 100 °C for 15 min and centrifuged. The concentrations 3-idoloacetic acid bound to BSA and adsorbed by MOF was determined by Knauer HPLC-UV-Vis chromatography (KNAUER C18 HPLC column, 5 µm particle, 100A, 150 × 4.6 mm^2 I.D.) in reverse phase by using isocratic elution with the mobile phase of 5 mM NaH_2PO_4 and acetonitrile (90:10 v/v %). The flow rate was adjusted at 1 ml/min, and a detection wavelength of 274 nm was used. To determine the amount of 3-indoloacetic acid bound to BSA, the blind trial without UiO-66-NH_2 (75%) was performed as above. The structure of the BSA depicted in Figure 7D was adapted from Abdelhameed, R. M. et al., 2018 [12].

The recyclability of UiO-66 12.5% HCl, UiO-66-NH_2 (75%) and UiO-66-NH_2 (75%) 12.5% HCl for the adsorption of 3-indoloacetic acid was performed as above. After each adsorption, the MOF sample was filtered and dried overnight. The equilibrium concentration of 3-indoloacetic acid was determined as described above. Prior to the next sorption, the sample was activated in methanol in ultrasound bath for 5 min, centrifuged and subsequently dried

overnight. Finally, the samples were activated under vacuum at 120 °C for 4 h. The three cycles of sorption-desorption were performed.

Results

PXRD

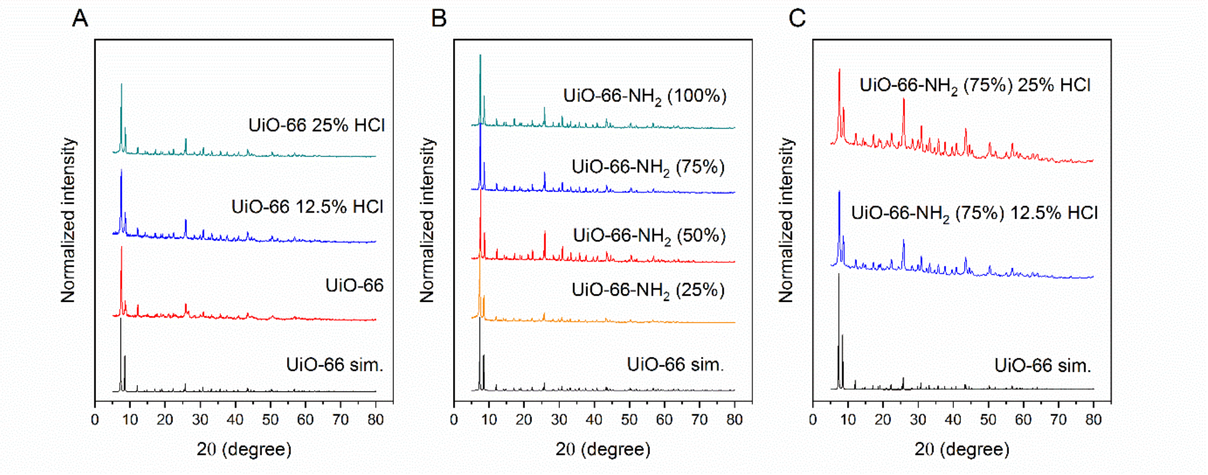

Figure S1. PXRD results of prepared UiO-66 samples; A) pristine UiO-66 and defective UiO-66 12.5% HCl and UiO-66 25% HCl; B) series of UiO-66-NH_2 samples; C) defective UiO-66-NH_2 samples

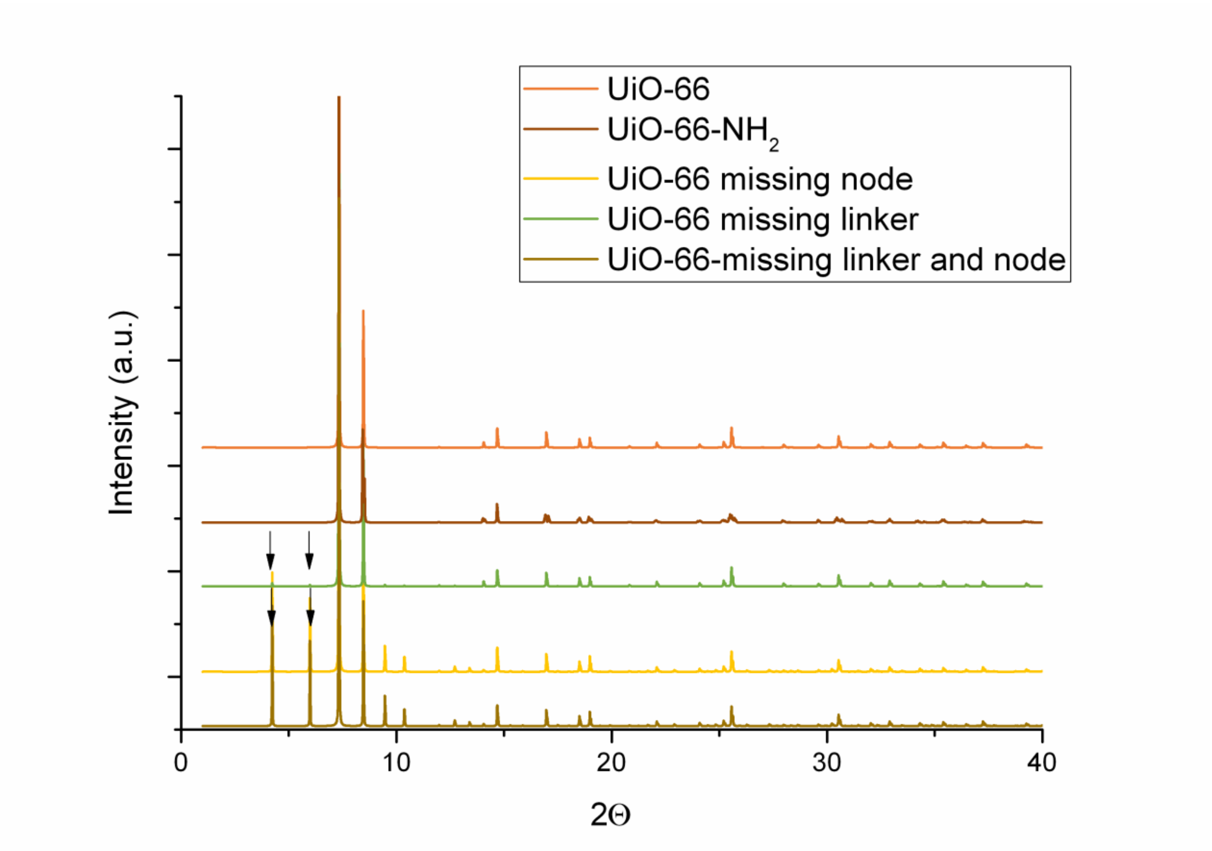

Figure S2. Simulated XRD patterns for prepared UiO-66 samples; additional reflections in defective UiO-66 samples marked with arrows; PXRD pattern of UiO-66 and UiO-66-NH_2 simulated from modified .cif files

UV-Vis

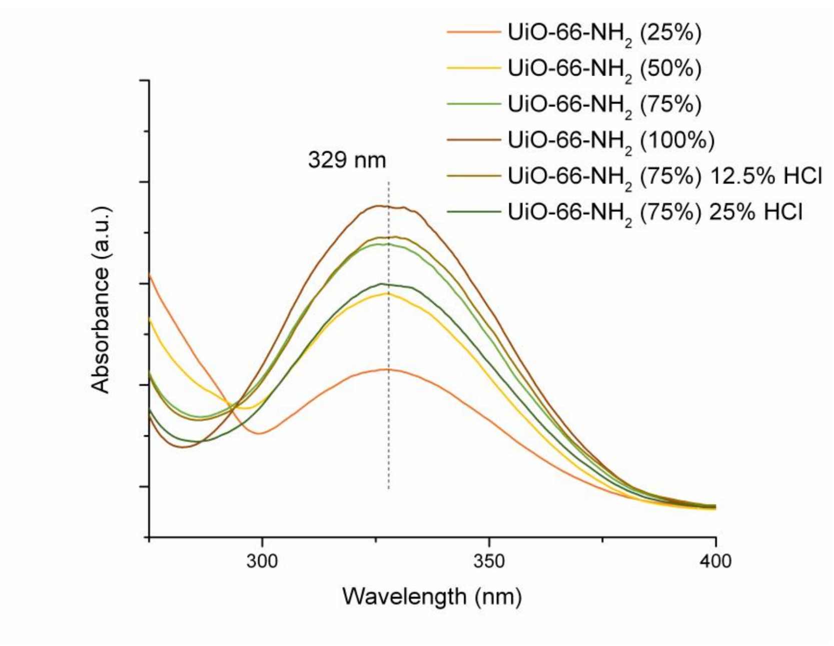

Figure S3. UV-Vis absorbance spectra of UiO-66-NH_2 (X%) and UiO-66-NH_2 (X%) Y% HCl samples diluted in 1 M $NaOH_{(aq.)}$ used for determination of H_2BDC-NH_2

Low temperature N_2 sorption

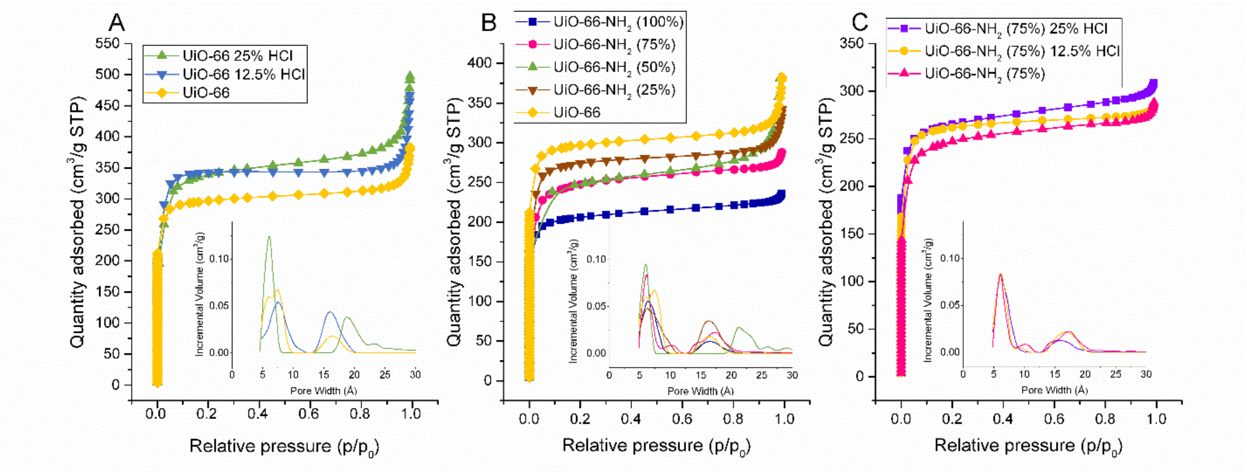

Figure S4. Low temperature N_2 sorption isotherms of prepared UiO-66 samples and pore size distribution (insets); A) pristine UiO-66 and defective UiO-66 12.5% HCl and UiO-66 25% HCl; B) series of UiO-66-NH_2 samples; C) defective UiO-66-NH_2 samples

TGA/DSC

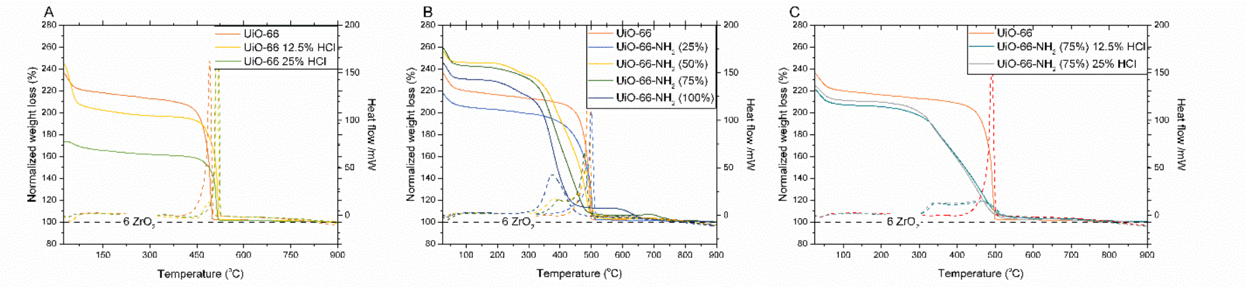

Figure S5. TGA/DSC results of prepared UiO-66 samples; A) pristine UiO-66 and defective UiO-66 12.5% HCl and UiO-66 25% HCl; B) series of UiO-66-NH_2 samples; C) defective UiO-66-NH_2 samples; TGA results-solid lines; DSC results- dashed lines

In situ DRIFT

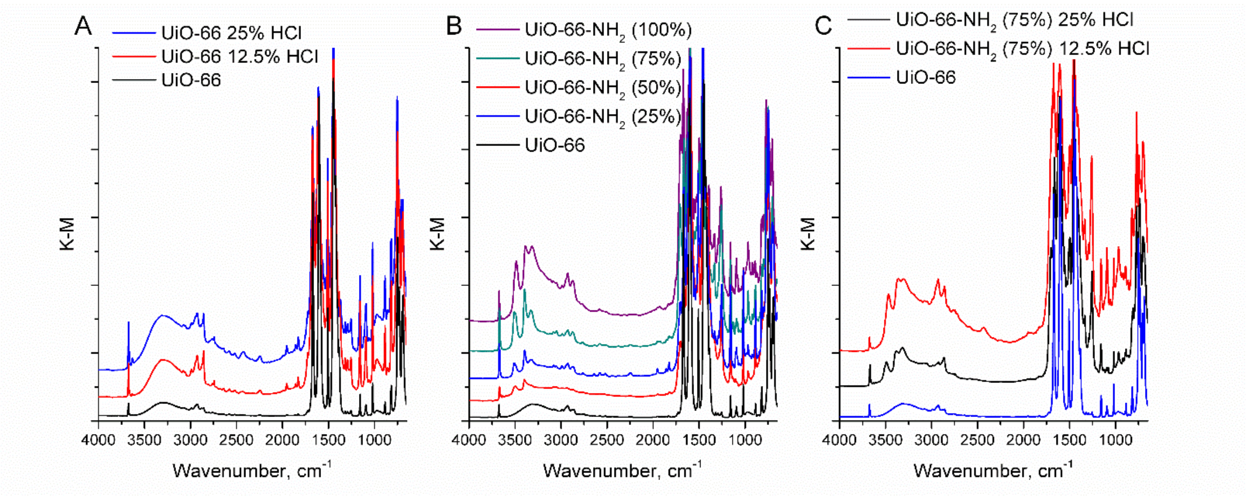

Figure S6. *In situ* DRIFT analysis of prepared samples; A) pristine UiO-66 and defective UiO-66 12.5% HCl and UiO-66 25% HCl; B) series of UiO-66-NH_2 samples; C) defective UiO-66-NH_2 samples

The bands characteristic at 1610, 1446, 1367, 883 and 746 cm^{-1} correspond to ν_{as} -OCO, ν_s -OCO, CC ring vibrations, -OH + CH bending vibrations (antiphase) and -OH + CH bending vibrations (in-phase), [13] respectively. Additional sharp, intense band at 3670 cm^{-1} originates from μ_3-OH groups from zirconium clusters. [13,14] The bands in the 3100-2750 cm^{-1} range are attributed to ν -CH vibrations originating from the organic linker. [14]

Dissolution ^{1}H NMR

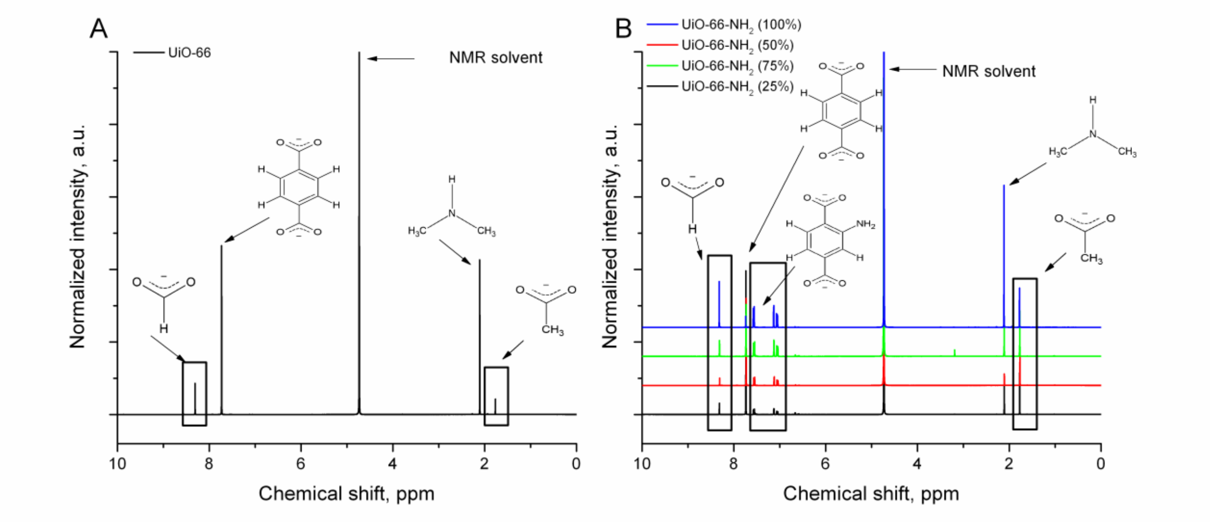

Figure S7. Dissolution ^{1}H NMR spectra of as-received UiO-66 samples prepared using acetic acid as a modulator; A) pristine UiO-66 and defective UiO-66 12.5% HCl and UiO-66 25% HCl; B) series of UiO-66-NH_2 samples

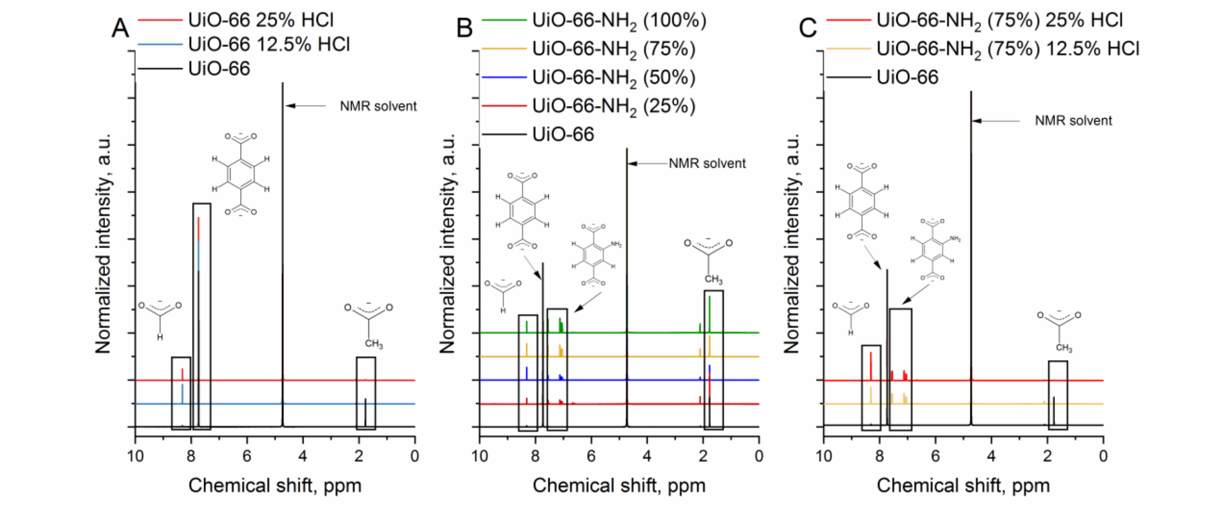

Figure S8. Dissolution ^{1}H NMR spectra of activated samples; A) pristine UiO-66 and defective UiO-66 12.5% HCl and UiO-66 25% HCl; B) series of UiO-66-NH_2 samples; C) defective UiO-66-NH_2 samples

DR UV-Vis

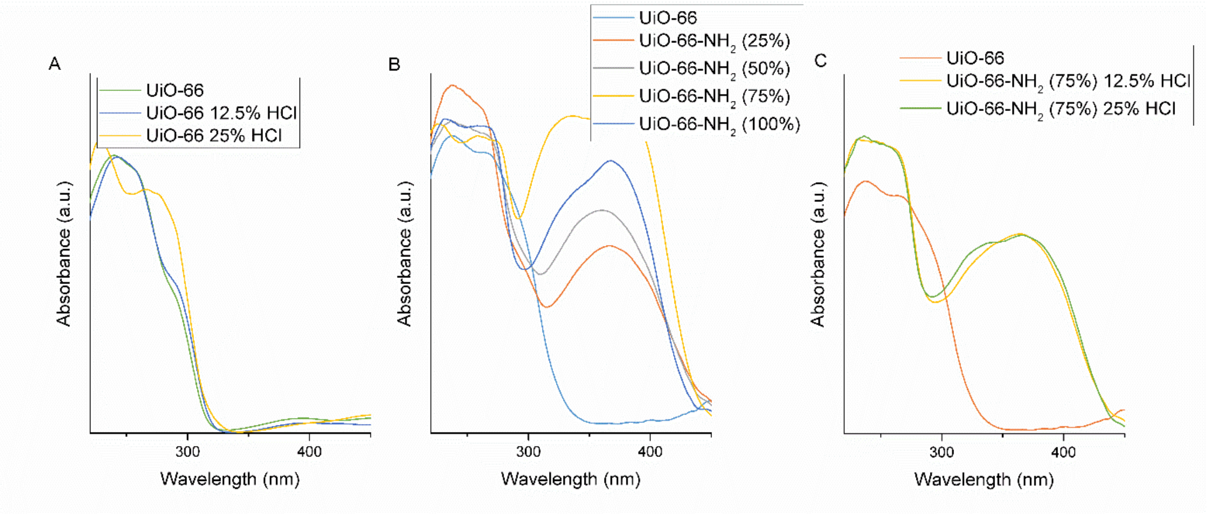

Figure S9. DR UV-Vis spectra of A) pristine UiO-66 and defective UiO-66 12.5% HCl and UiO-66 25% HCl; B) series of UiO-66-NH_2 samples; C) defective UiO-66-NH_2 samples

The DR UV-Vis spectra performed for powder UiO-66 materials exhibit a wide band in 200-310 nm range. The band at approx. 300 nm originates from $\pi \rightarrow \pi^*$transitions of the aromatic ring from organic linker. [13] The spectra of UiO-66-NH_2 samples prepared by the mixed-linker route exhibit additional bands at approx. 370 nm, which is attributed to $n \rightarrow \pi^*$ transitions of H_2BDC-NH_2 responsible for the yellow color of the organic linker and the corresponding UiO-66-NH_2. The spectra of UiO-66-NH_2 samples prepared by mixed-linker exhibit a considerable decrease of the 370 nm band with the decrease of H_2BDC-NH_2, which supports the quantitative UV-Vis results.

DLS

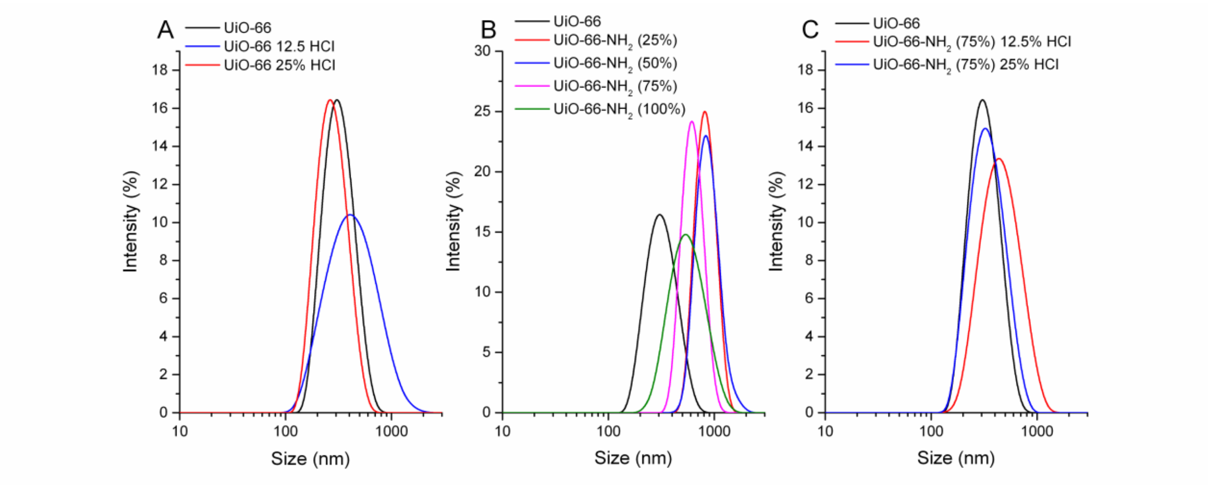

Figure S10. Hydrodynamic particle size distribution of A) pristine UiO-66 and defective UiO-66 12.5% HCl and UiO-66 25% HCl; B) series of UiO-66-NH_2 samples; C) defective UiO-66-NH_2 samples

Adsorption of uremic toxins

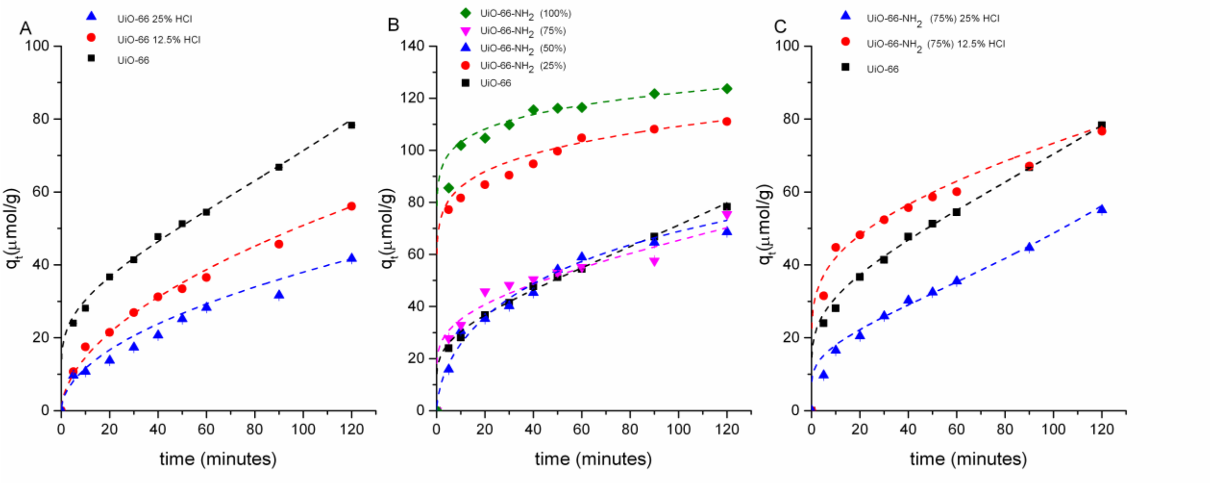

Figure S11. Adsorption efficiency of 1mg of prepared UiO-66 samples in 1.5 mL 0.1 mM hippuric acid as a function of time (293 K); A) pristine UiO-66 and defective UiO-66 12.5% HCl and UiO-66 25% HCl; B) series of UiO-66-NH_2 samples; C) defective UiO-66-NH_2 samples

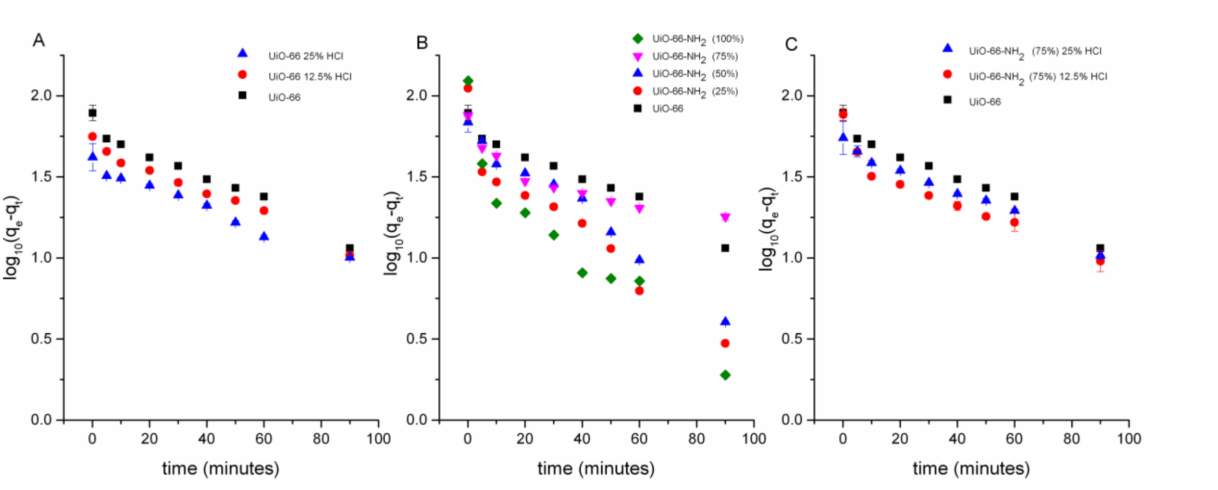

Figure S12. Pseudo-first-order kinetic model of adsorption of hippuric acid on prepared UiO-66 samples; 1mg of prepared UiO-66 samples in 1.5 mL 0.1mM hippuric acid as a function of time (293 K); A) pristine UiO-66 and defective UiO-66 12.5% HCl and UiO-66 25% HCl; B) series of UiO-66-NH_2 samples; C) defective UiO-66-NH_2 samples

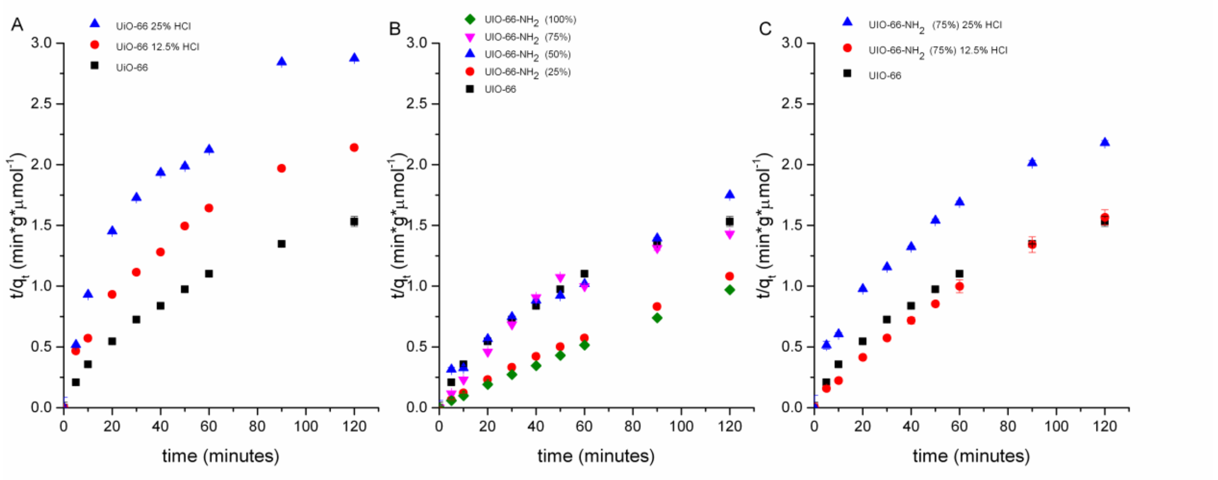

Figure S13. Pseudo-second-order kinetic model of adsorption of hippuric acid on prepared UiO-66 samples; 1mg of prepared UiO-66 samples in 1.5 mL 0.1 mM hippuric acid as a function of time (293 K); A) pristine UiO-66 and defective UiO-66 12.5% HCl and UiO-66 25% HCl; B) series of UiO-66-NH_2 samples; C) defective UiO-66-NH_2 samples

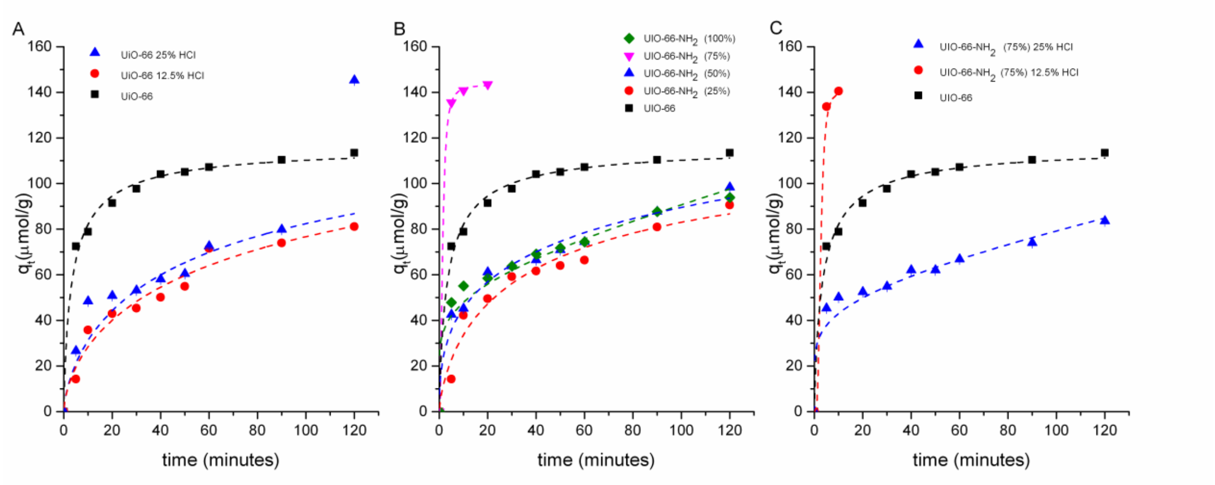

Figure S14. Adsorption efficiency of 1 mg of prepared UiO-66 samples in 1.5 mL 0.1 mM 3-indoloacetic acid as a function of time (293 K); A) pristine UiO-66 and defective UiO-66 12.5% HCl and UiO-66 25% HCl; B) series of UiO-66-NH_2 samples; C) defective UiO-66-NH_2 samples

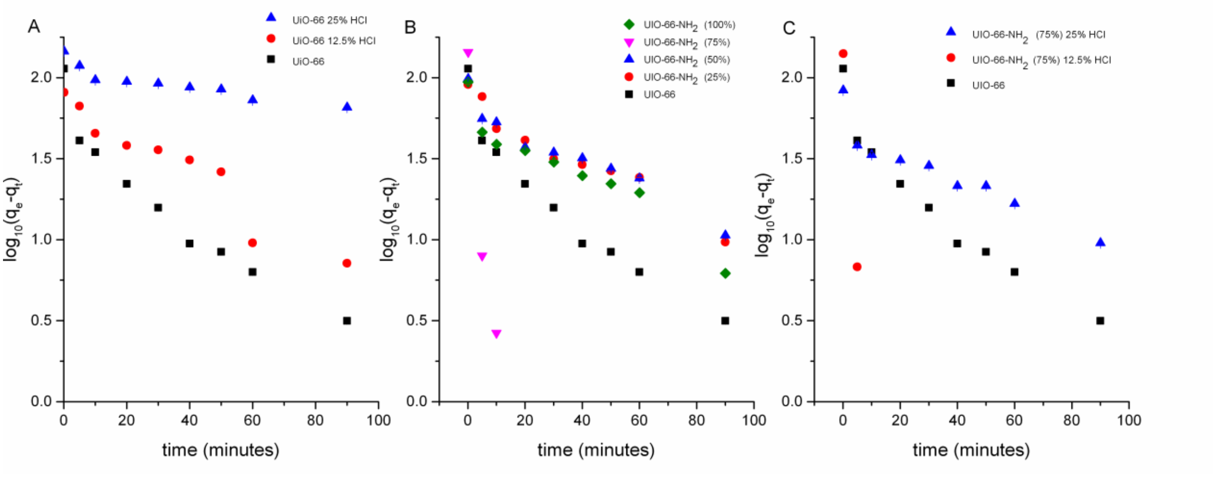

Figure S15. Pseudo-first-order kinetic model of adsorption of 3-indoloacetic acid on prepared UiO-66 samples; 1mg of prepared UiO-66 samples in 1.5 mL 0.1 mM 3-indoloacetic acid as a function of time (293 K); A) pristine UiO-66 and defective UiO-66 12.5% HCl and UiO-66 25% HCl; B) series of UiO-66-NH_2 samples; C) defective UiO-66-NH_2 samples

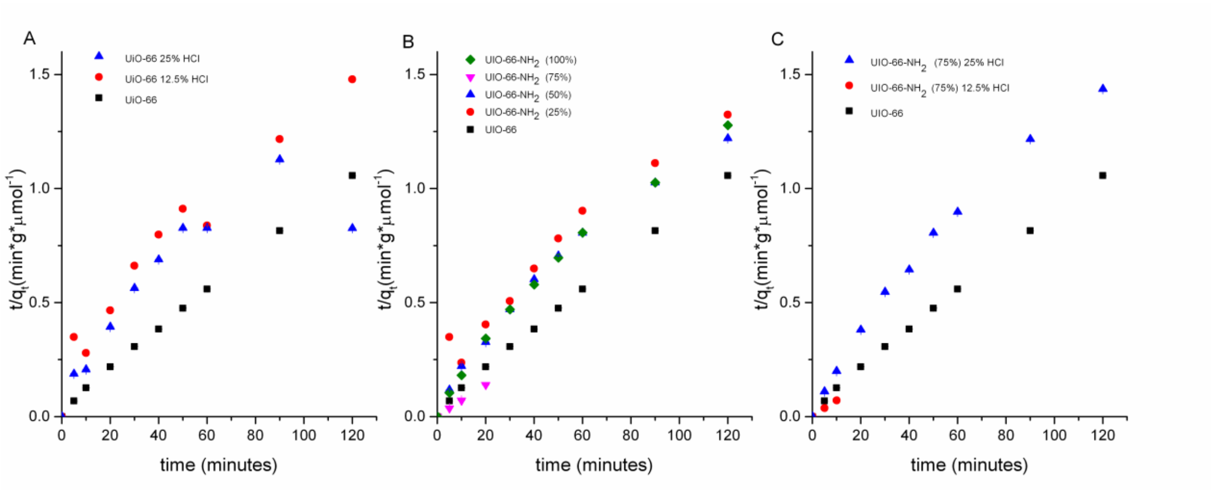

Figure S16. Pseudo-second-order kinetic model of adsorption of 3-indoloacetic acid on prepared UiO-66 samples; 1 mg of prepared UiO-66 samples in 1.5 mL 0.1 mM 3-indoloacetic acid as a function of time (293 K); A) pristine UiO-66 and defective UiO-66 12.5% HCl and UiO-66 25% HCl; B) series of UiO-66-NH_2 samples; C) defective UiO-66-NH_2 samples

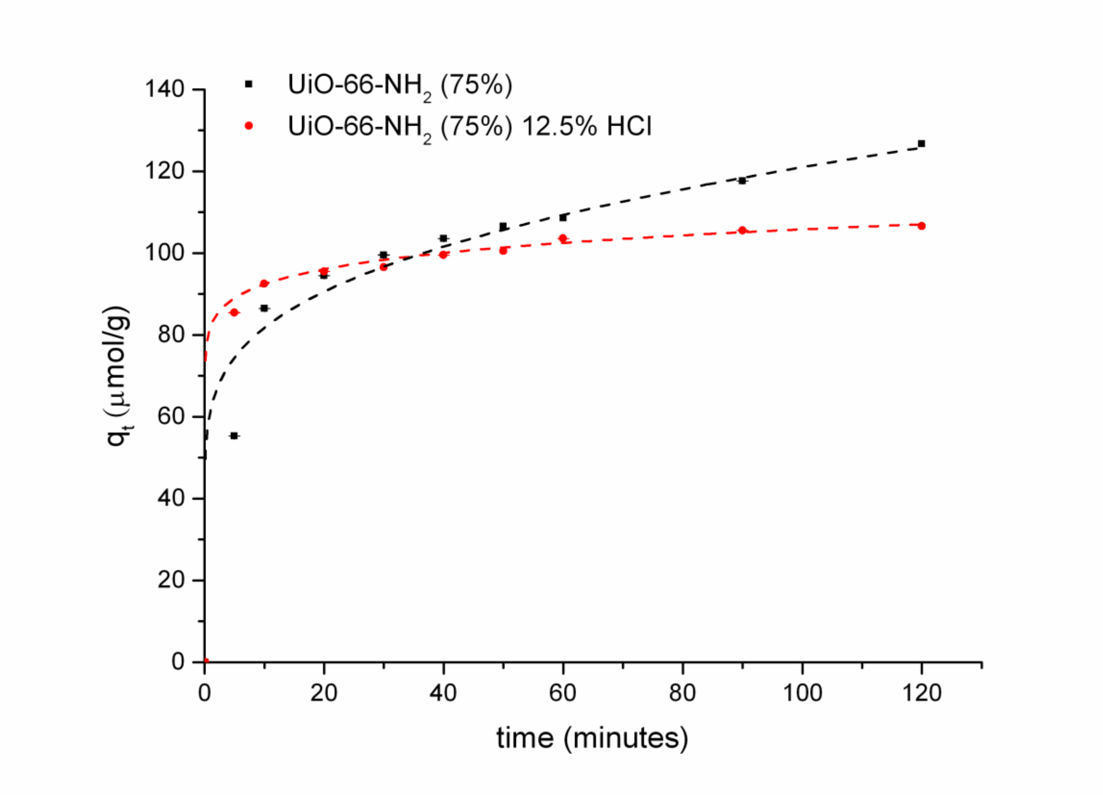

Figure S17. Adsorption efficiency of 0.5 mg of prepared UiO-66-NH_2 (75%) and UiO-66-NH_2 (75%) 12.5% HCl samples in 1.5 mL 0.1 mM 3-indoloacetic acid as a function of time (293 K)

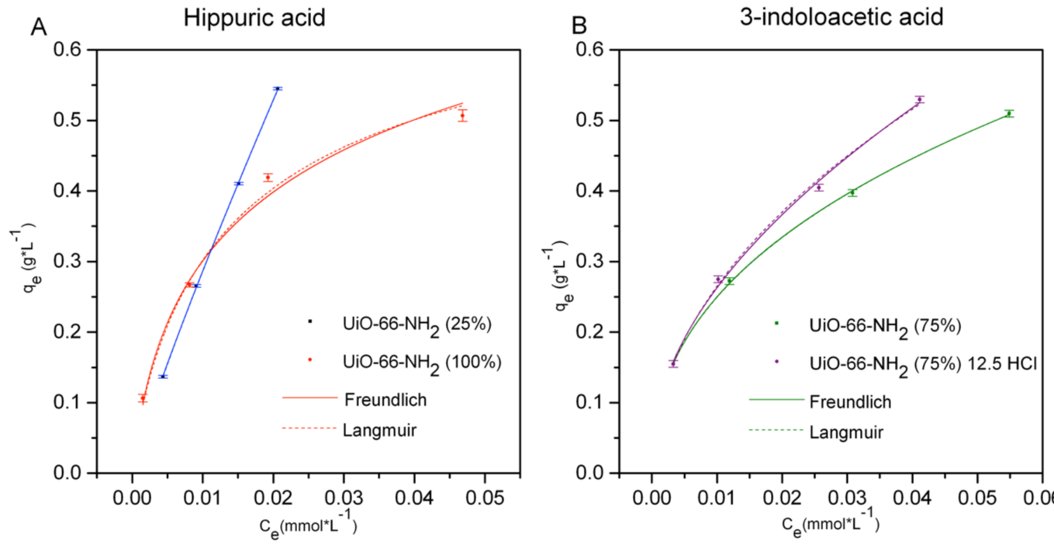

Figure S18. Adsorption isotherms of A) hippuric acid, B) 3-indoloaceic acid for selected UiO-66 samples; solid line – Freundlich isotherm, dashed line – Langmuir isotherm

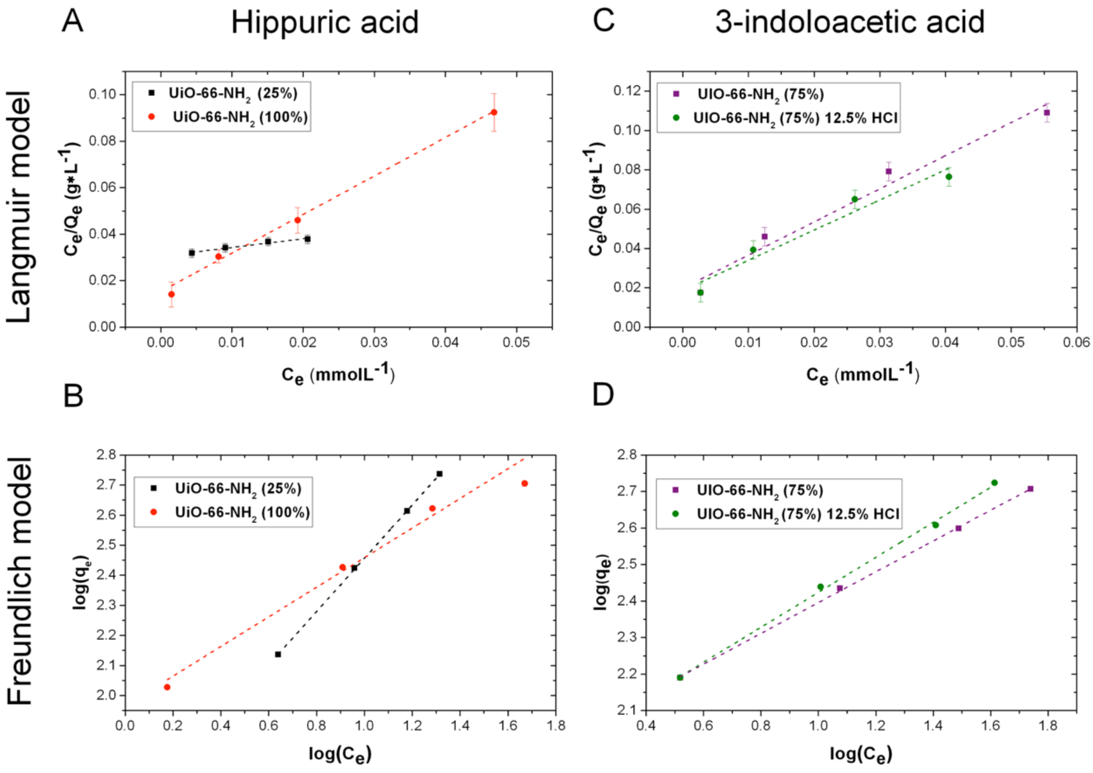

Figure S19. Langmuir and Freundlich plots for A) and B) hippuric acid and C) and D) 3-indoloacetic acid for selected UiO-66 samples

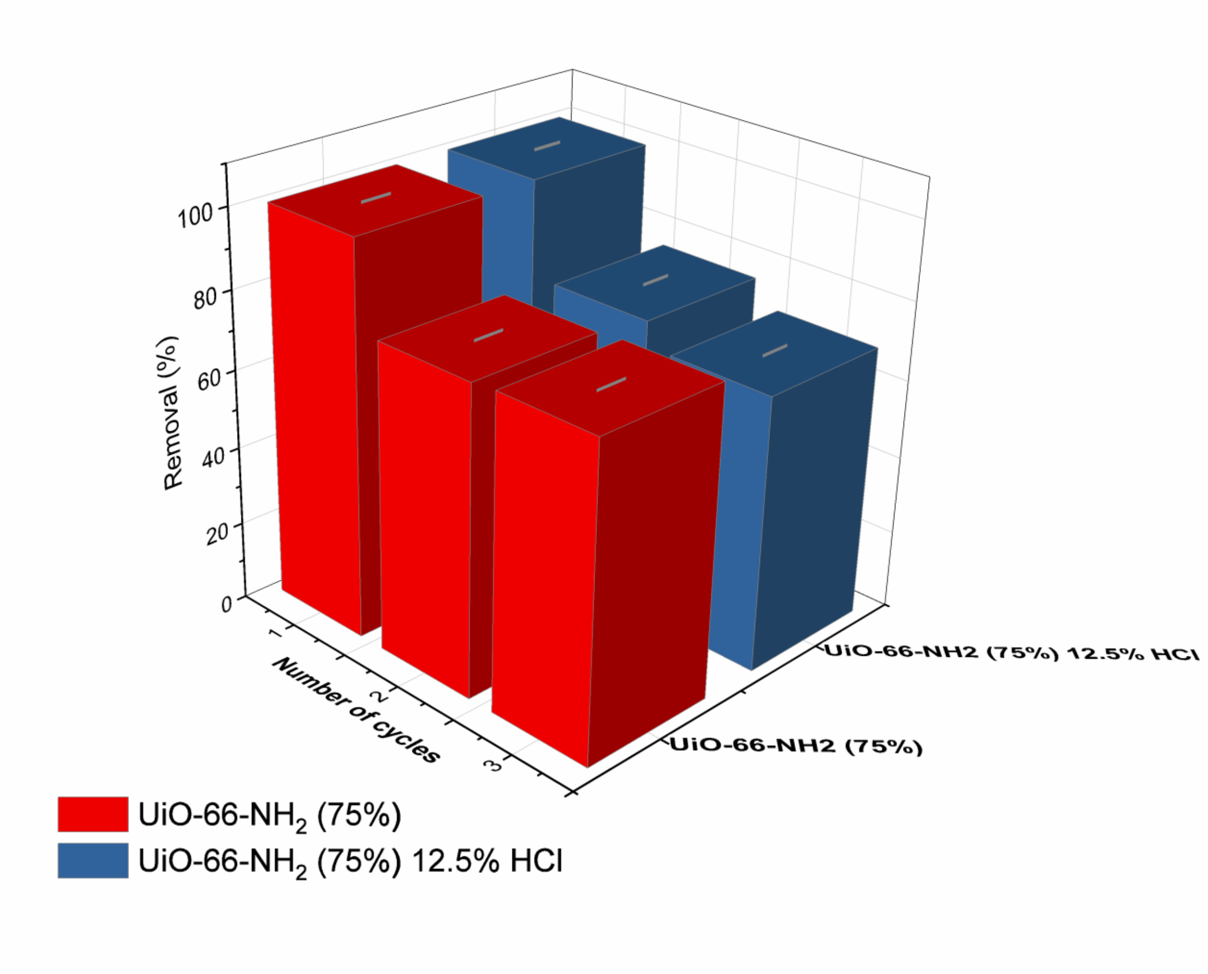

Figure S20. 3-indoloacetic acid adsorption recyclability; 1mg of prepared UiO-66 samples in 1.5 mL 0.1 mM 3-indoloacetic acid as a function of time (293 K)

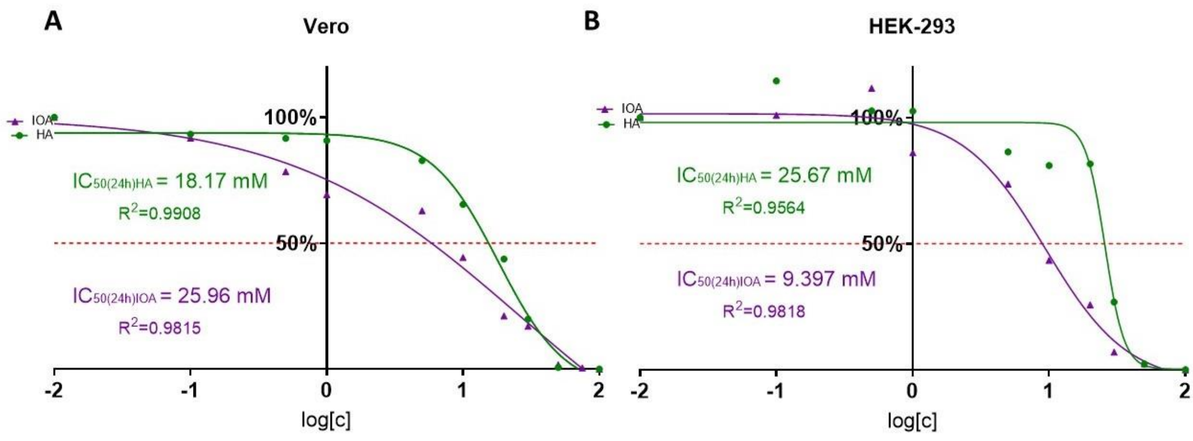

Figure S21. A) Evaluation of IC50 for hippuric and 3-indoleacetic acids on Vero cell line; B) Evaluation of IC_{50} for hippuric acid and 3-indoleacetic acid on HEK-293 cell line

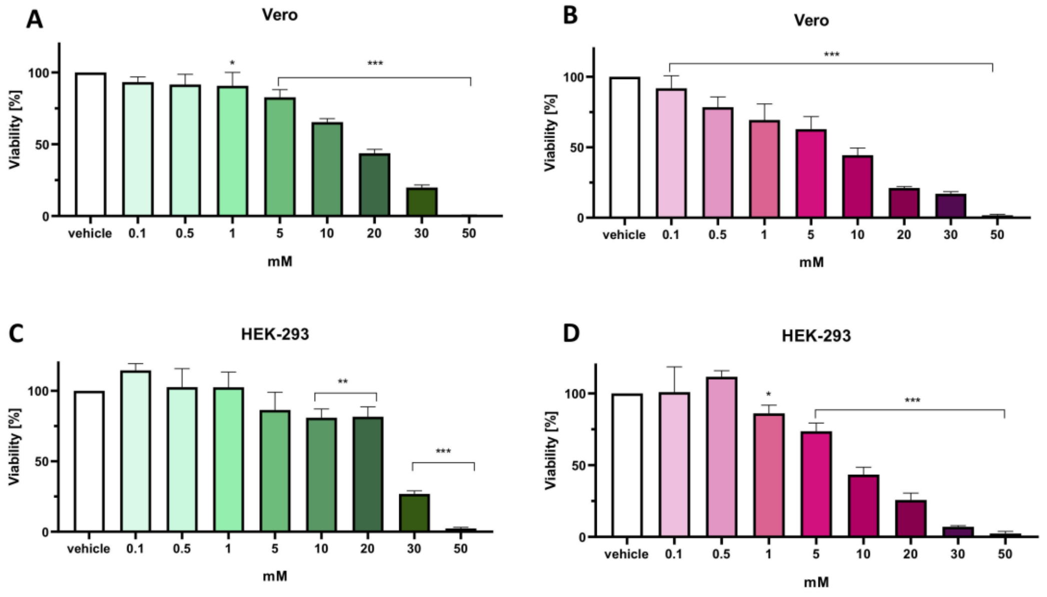

Figure S22. Viability (%) of hippuric acid (A) and 3-indoleacetic acid (B) on Vero cell line; Viability (%) of hippuric acid (C) and 3-indoleacetic acid (D) on HEK-293 cell line, mean values±SD, *p<0.01, **p<0.05, ***p<0.001, Tukey test.

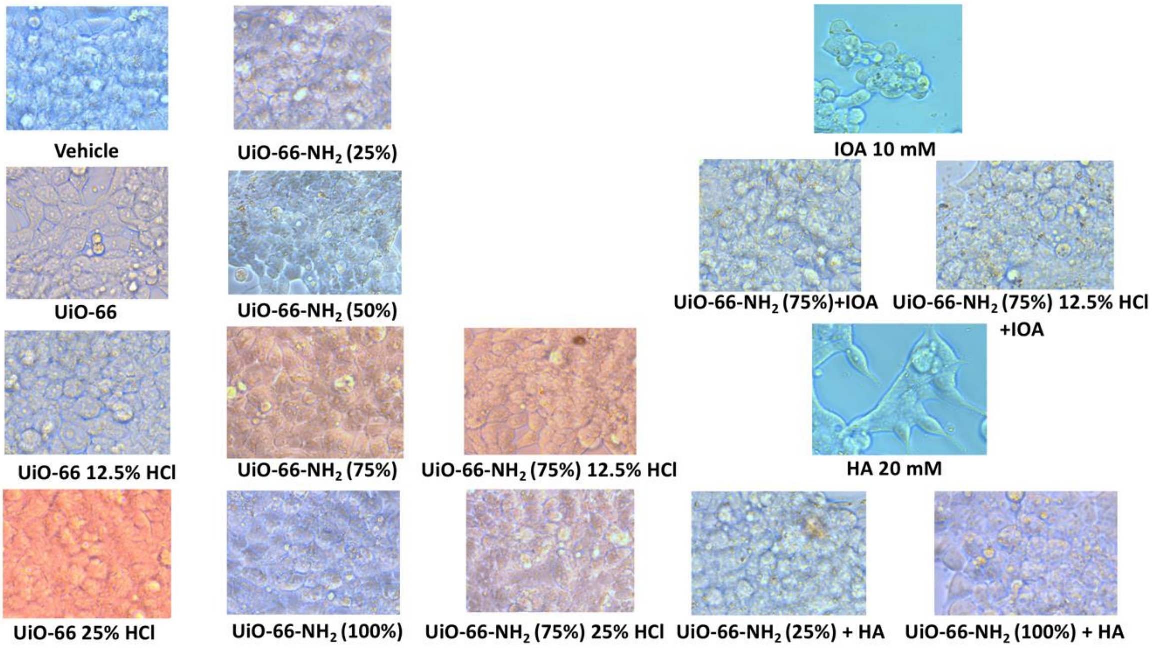

Figure S23. Images of HEK-293 cells after 24 h treatment with UiO-66 samples without and with hippuric and 3-indoloacetic acids.

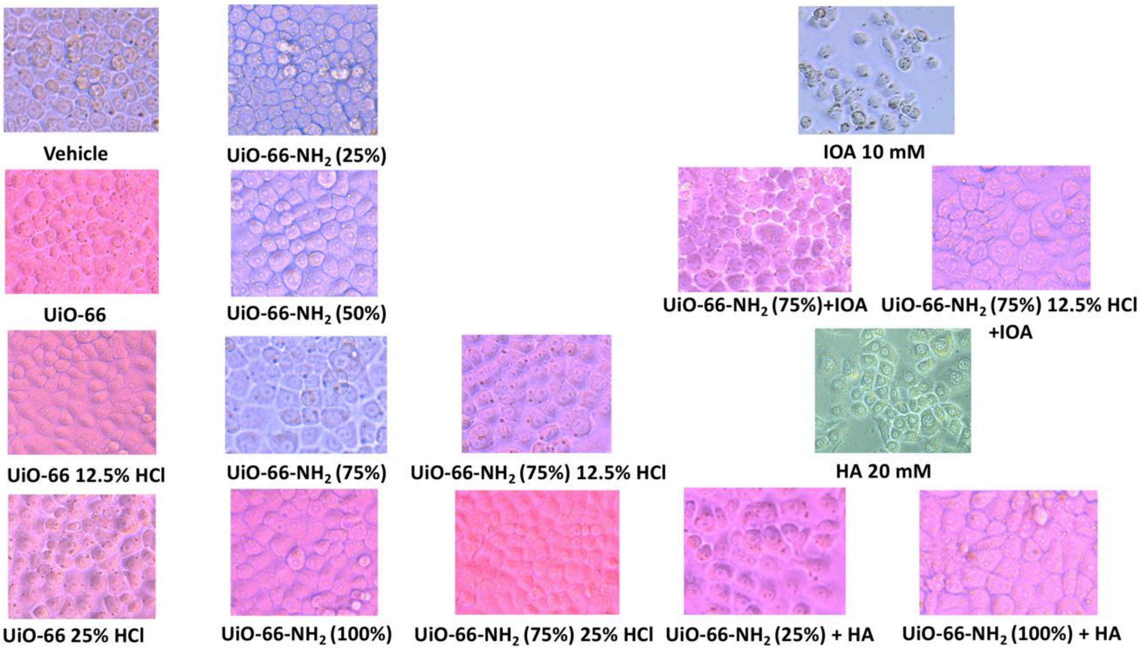

Figure S24. Images of Vero cells after 24 h treatment with UiO-66 samples without and with hippuric and 3-indoloacetic acids.

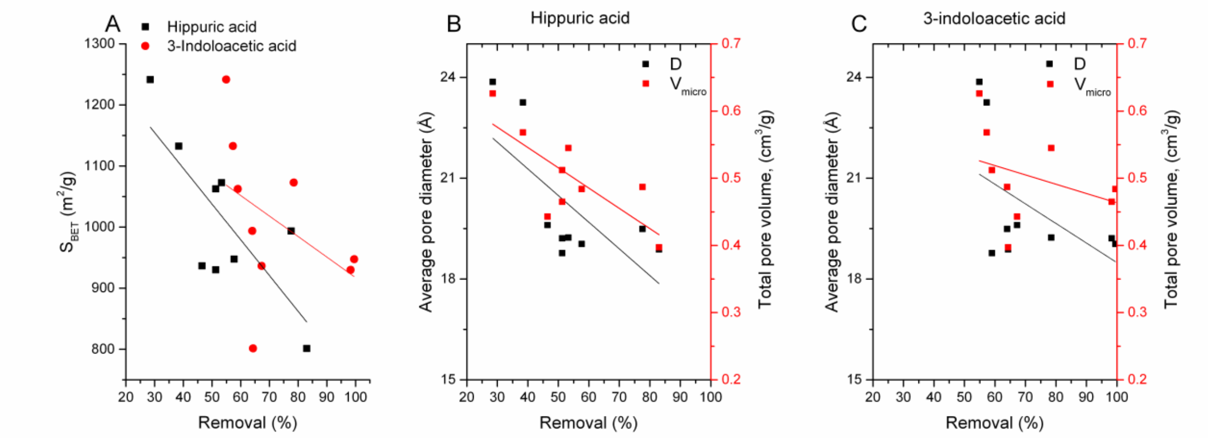

Figure S25. Correlations between toxin removal efficiencies with structural parameters: A) hippuric acid and 3-indoloacetic acid removal efficiencies with BET surface area; B) hippuric acid and C) 3-indoloacetic acid removal efficiencies with average pore diameter and total pore volume

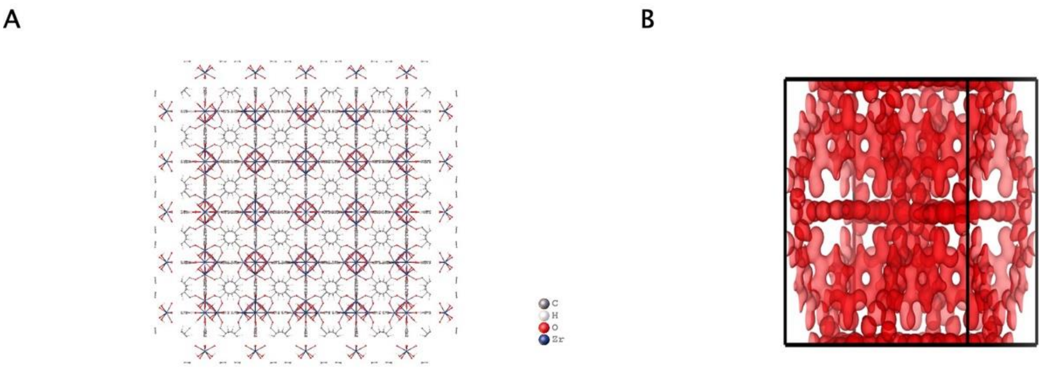

Figure S26. A) Structural model of "defect free" UiO-66; B) simulated model electron densities

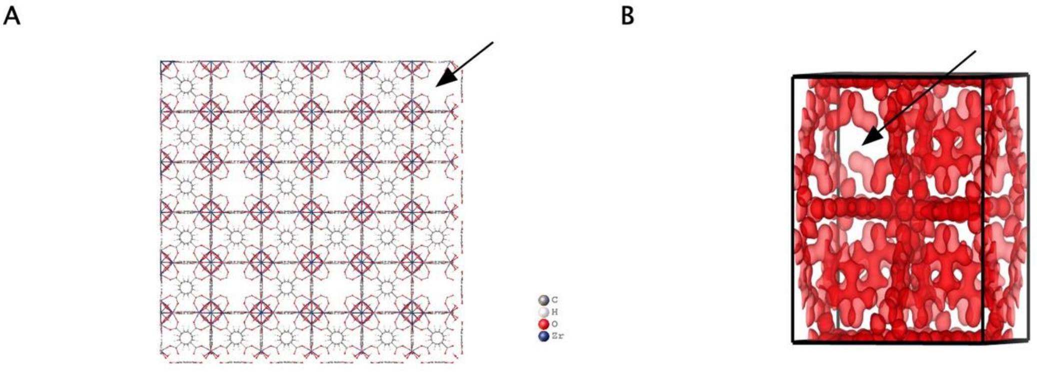

Figure S27. A) Structural model of UiO-66 missing liker regions; B) simulated model electron densities; missing linker marked with arrows

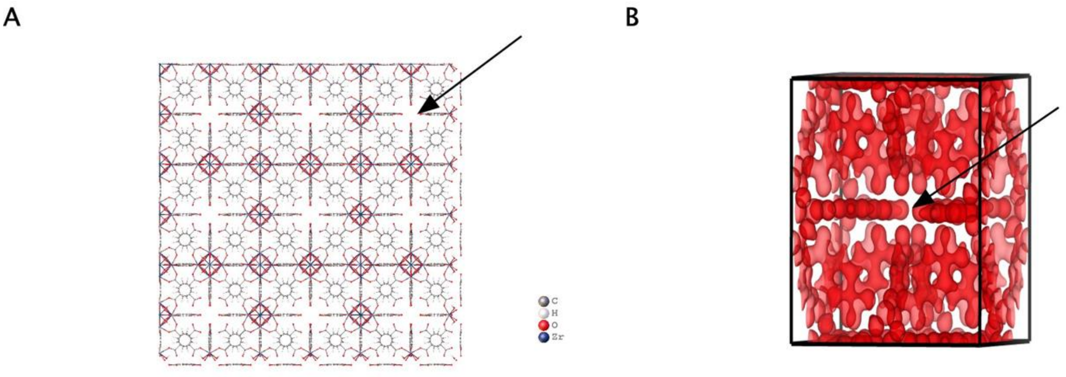

Figure S28. A) Structural model of UiO-66 missing node regions; B) simulated model electron densities; missing node marked with arrows

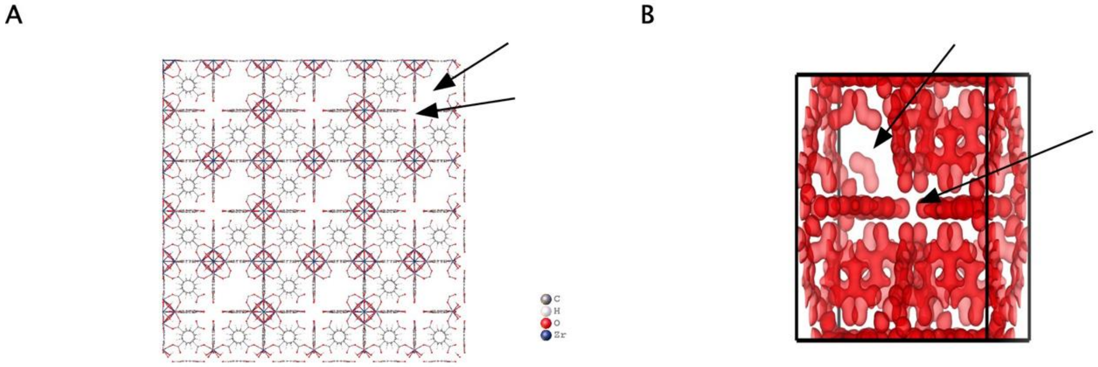

Figure S29. A) Structural model of UiO-66 missing linker and missing node regions; B) simulated model electron densities; missing linker and missing node regions marked with arrows

Table S1. Synthesis details of mixed linker-defective UiO-66 samples

Sample	$ZrCl_4$	H_2BDC	$H_2BDC-NH_2$	DMF	AcOH	HCl
	mmol	mmol	mmol	ml	ml	ml
UiO-66	0.82	0.78	X	81.7	9.2	x
UiO-66 12.5% HCl	1.59	1.57	X	43.75	x	6.25
UiO-66 25% HCl	1.59	1.57	X	37.5	x	12.5
UiO-66-NH_2 (25%)	0.82	0.60	0.19	81.7	9.2	x
UiO-66-NH_2 (50%)	0.82	0.40	0.40	81.7	9.2	x
UiO-66-NH_2 (75%)	0.82	0.20	0.55	81.7	9.2	x
UiO-66-NH_2 (100%)	0.82	x	0.72	81.7	9.2	x
UiO-66-NH_2 (75%) 12.5% HCl	1.59	0.39	1.08	43.75	x	6.25
UiO-66-NH_2 (75%) 25% HCl	1.59	0.39	1.08	37.5	x	12.5

Table S2. UV-Vis quantitative analysis results of $H_2BDC-NH_2$

Sample	Sample mass [mg]	Actual mass of MOF, [mg]	Calc. mass of $H_2BDC-NH_2$, [mg]	Calc. [$H_2BDC-NH_2 \times 10^{-4}$], mol/dm^3	Exp. [$H_2BDC-NH_2 \times 10^{-4}$] mol/dm^3	Exp. mol % of $H_2BDC-NH_2$
UiO-66-NH_2 (25%)	20	14.00	2.23	3.52	4.57	32
UiO-66-NH_2 (50%)	20	14.00	4.41	6.96	6.88	49
UiO-66-NH_2 (75%)	20	14.00	6.53	10.30	9.35	68
UiO-66-NH_2 (100%)	19,9	13.93	8.55	13.49	11.02	82
UiO-66-NH_2 (75%) 12.5% HCl	19,9	13.93	6.50	10.25	9.73	71
UiO-66-NH_2 (75%) 25% HCl	20.0	14.00	6.53	10.30	7.10	52

Table S3. Acetate to BDC, formate to BDC, and total modulator to molar ratios in prepared samples obtained from digestion/liquid ^{1}H NMR spectroscopy

Sample	$\left(\frac{Acetate}{BDC} m_R\right)$	$\left(\frac{Formate}{BDC} m_R\right)$	$\left(\frac{Total\ Mod.}{BDC} m_R\right)^*$
UiO-66	0.16	0.02	0.18
UiO-66 12.5% HCl	0.00	0.30	0.30
UiO-66 25% HCl	0.00	0.18	0.18
UiO-66-NH_2 (25%)	0.19	0.10	0.29
UiO-66-NH_2 (50%)	0.10	0.23	0.33
UiO-66-NH_2 (75%)	0.22	0.29	0.51
UiO-66-NH_2 (100%)	0.33	0.28	0.61
UiO-66-NH_2 (75%) 12.5% HCl	0.00	0.38	0.38
UiO-66-NH_2 (75%) 25% HCl	0.00	0.61	0.61

* Total modulator= sum of acetate and formate integrals from digestion/liquid ^{1}H NMR

Table S4. Kinetic parameters of pseudo-first order and pseudo-second order models for hippuric acid

Sample	Pseudo-first order			Pseudo-second order		
	k_1 (min^{-1})	q_e (µmol.g^{-1})	R^2	k_2 (g.µmol^{-1}.min^{-1})	q_e (µmol.g^{-1})	R^2
UiO-66	1.865E-02	66.07	0.97	6.000E-04	83.33	0.92
UiO-66 12.5% HCl	1.704E-02	50.12	0.98	6.283E-04	58.82	0.89
UiO-66 25% HCl	1.543E-02	37.15	0.98	6.914E-04	45.45	0.85

UiO-66-NH_2 (25%)	3.455E-02	56.23	0.91	2.031E-03	112.4	0.99
UiO-66-NH_2 (50%)	2.994E-02	63.10	0.98	7.348E-03	76.92	0.96
UiO-66-NH_2 (75%)	1.405E-02	50.12	0.80	1.225E-03	71.43	0.95
UiO-66-NH_2 (100%)	3.685E-02	51.29	0.88	3.048E-03	125.0	0.99
UiO-66-NH_2 (75%) 12.5% HCl	1.935E-02	48.98	0.89	1.300E-03	76.92	0.98
UiO-66-NH_2 (75%) 25% HCl	1.704E-02	50.12	0.98	5.898E-04	58.82	0.88

Table S5. Kinetic parameters of pseudo-first order and pseudo-second-order models for 3-indoloacetic acid

Sample	Pseudo-first order			Pseudo-second order		
	k_1 (min^{-1})	q_e ($\mu mol.g^{-1}$)	R^2	k_2 ($g.\mu mol^{-1}.min^{-1}$)	q_e ($\mu mol.g^{-1}$)	R^2
UiO-66	3.685E-02	54.95	0.91	2.294E-03	114.9	0.99
UiO-66 12.5% HCl	2.764E-02	74.13	0.92	5.261E-04	90.91	0.94
UiO-66 25% HCl	7.370E-03	120.2	0.84	2.700E-04	126.6	0.73
UiO-66-NH_2 (25%)	2.211E-02	74.13	0.94	5.263E-04	100.00	0.95
UiO-66-NH_2 (50%)	2.050E-02	70.79	0.93	9.912E-04	90.83	0.96
UiO-66-NH_2 (75%)	3.915E-01	107.1	0.94	4.455E-02	142.8	0.99
UiO-66-NH_2 (100%)	2.395E-02	64.57	0.91	1.075E-03	95.24	0.97
UiO-66-NH_2 (75%) 12.5% HCl	5.988E-01	141.2	0.99	1.260E-01	140.8	0.99
UiO-66-NH_2 (75%) 25% HCl	1.911E-02	51.29	0.87	1.309E-03	83.33	0.97

Table S6. Langmuir and Freundlich parameters for uremic toxins adsorption on prepared MOF samples

Sample	Langmuir model			Freundlich model		
	$k_{L,}$ (L.µmol^{-1})	q_{max} (µmol.g^{-1})	R^2	$k_{F,}$ (µmol^{n-1}L^{n}g^{-1})	n	R^2
**Hippuric acid**						
UiO-66-NH_2 (25%)	12.24	2.670	0.97	37.24	1.128	0.99
UiO-66-NH_2 (100%)	84.05	0.856	0.99	93.58	2.132	0.98
**3-indoloacetic acid**						
UiO-66-NH_2 (75%)	77.87	0.609	0.98	94.30	2.371	0.99
UiO-66-NH_2 (75%) 12.5% HCl	73.80	0.674	0.97	88.17	2.088	0.99

Table S7. Mean viability (%) of HaKaT, Vero and HEK-293 cell after 24h incubation with UiO-66 determined by MTT test.

**Sample**	**Viability % HaKaT**	**Viability % Vero**	**Viability % HEK-293**
UiO-66	101.673	99.949	100.924
UiO-66 12.5%HCl	97.972	98.350	104.208
UiO-66 25% HCl	101.621	104.269	113.064
UiO-66-NH_2 (25%)	99.692	106.252	117.610
UiO-66-NH_2 (50%)	98.188	105.854	115.722
UiO-66-NH_2 (75%)	97.562	106.896	112.018
UiO-66-NH_2 (100%)	96.338	85.941	105.737
UiO-66-NH_2 (75%) 12.5% HCl	99.633	99.190	120.310
UiO-66-NH_2 (75%) 25% HCl	97.562	91.179	119.335
**IOA (10mM)**	**56.515**	**62.854**	**43.408**
UiO-66-NH2 (75%) + IOA	95.612	96.172	98.573

UiO-66-NH_2 (75%) 12.5% HCl + IOA	96.772	98.495	104.177
**HA (20 mM)**	**42.114**	**43.705**	**51.675**
UiO-66-NH_2 (25%) + HA	92.739	92.236	99.025
UiO-66-NH_2 (100%) + HA	94.729	97.149	95.413

REFERENCES

(1) Qiu, J.; Feng, Y.; Zhang, X.; Jia, M.; Yao, J. Acid-Promoted Synthesis of UiO-66 for Highly Selective Adsorption of Anionic Dyes: Adsorption Performance and Mechanisms. *J. Colloid Interface Sci.* **2017**, *499*, 151–158. https://doi.org/10.1016/j.jcis.2017.03.101.

(2) Clark, C. A.; Heck, K. N.; Powell, C. D.; Wong, M. S. Highly Defective UiO-66 Materials for the Adsorptive Removal of Perfluorooctanesulfonate. *ACS Sustain. Chem. Eng.* **2019**, *7* (7), 6619–6628. https://doi.org/10.1021/acssuschemeng.8b05572.

(3) Jodłowski, P. J.; Kurowski, G.; Kuterasiński, Ł.; Sitarz, M.; Jeleń, P.; Jaśkowska, J.; Kołodziej, A.; Pajdak, A.; Majka, Z.; Boguszewska-Czubara, A. Cracking the Chloroquine Conundrum: The Application of Defective UiO-66 Metal–Organic Framework Materials to Prevent the Onset of Heart Defects—In Vivo and In Vitro. *ACS Appl. Mater. Interfaces* **2021**, *13*, 312–323. https://doi.org/10.1021/acsami.0c21508.

(4) Øien, S.; Wragg, D.; Reinsch, H.; Svelle, S.; Bordiga, S.; Lamberti, C.; Lillerud, K. P. Detailed Structure Analysis of Atomic Positions and Defects in Zirconium Metal-Organic Frameworks. *Cryst. Growth Des.* **2014**, *14* (11), 5370–5372. https://doi.org/10.1021/cg501386j.

(5) Ni, B.; Sun, W.; Kang, J.; Zhang, Y. Understanding the Linear and Second-Order Nonlinear Optical Properties of UiO-66-Derived Metal-Organic Frameworks: A Comprehensive DFT Study. *J. Phys. Chem. C* **2020**, *124* (21), 11595–11608. https://doi.org/10.1021/acs.jpcc.0c01580.

(6) Momma, K.; Izumi, F. VESTA 3 for Three-Dimensional Visualization of Crystal, Volumetric and Morphology Data. *J. Appl. Crystallogr.* **2011**, *44* (6), 1272–1276. https://doi.org/10.1107/S0021889811038970.

(7) Dolomanov, O. V; Bourhis, L. J.; Gildea, R. J.; Howard, J. A. K.; Puschmann, H. OLEX2: A Complete Structure Solution, Refinement and Analysis Program. *J. Appl. Crystallogr.* **2009**, *42* (2), 339–341. https://doi.org/10.1107/S0021889808042726.

(8) Chavan, S. M.; Shearer, G. C.; Svelle, S.; Olsbye, U.; Bonino, F.; Ethiraj, J.; Lillerud, K. P.; Bordiga, S. Synthesis and Characterization of Amine-Functionalized Mixed-Ligand Metal-Organic Frameworks of UiO-66 Topology. *Inorg. Chem.* **2014**, *53* (18), 9509–9515. https://doi.org/10.1021/ic500607a.

(9) Shearer, G. C.; Chavan, S.; Bordiga, S.; Svelle, S.; Olsbye, U.; Lillerud, K. P. Defect Engineering: Tuning the Porosity and Composition of the Metal-Organic Framework UiO-66 via Modulated Synthesis. *Chem. Mater.* **2016**, *28* (11), 3749–3761. https://doi.org/10.1021/acs.chemmater.6b00602.

(10) Kato, S.; Otake, K.; Chen, H.; Akpinar, I.; Buru, C. T.; Islamoglu, T.; Snurr, R. Q.; Farha, O. K. Zirconium-Based Metal–Organic Frameworks for the Removal of Protein-Bound Uremic Toxin from Human Serum Albumin. *J. Am. Chem. Soc.* **2019**, *141*, 2568–2576. https://doi.org/10.1021/jacs.8b12525.

(11) Pankajakshan, A.; Sinha, M.; Ojha, A. A.; Mandal, S. Water-Stable Nanoscale

Zirconium-Based Metal-Organic Frameworks for the Effective Removal of Glyphosate from Aqueous Media. *ACS Omega* **2018**, *3* (7), 7832–7839. https://doi.org/10.1021/acsomega.8b00921.

(12) Bujacz, A. Structures of Bovine, Equine and Leporine Serum Albumin. *Acta Crystallogr. Sect. D* **2012**, *68* (10), 1278–1289. https://doi.org/10.1107/S0907444912027047.

(13) Valenzano, L.; Civalleri, B.; Chavan, S.; Bordiga, S.; Nilsen, M. H.; Jakobsen, S.; Lillerud, K. P.; Lamberti, C. Disclosing the Complex Structure of UiO-66 Metal Organic Framework: A Synergic Combination of Experiment and Theory. *Chem. Mater.* **2011**, *23* (7), 1700–1718. https://doi.org/10.1021/cm1022882.

(14) Chakarova, K.; Strauss, I.; Mihaylov, M.; Drenchev, N.; Hadjiivanov, K. Evolution of Acid and Basic Sites in UiO-66 and UiO-66-NH2 Metal-Organic Frameworks: FTIR Study by Probe Molecules. *Microporous Mesoporous Mater.* **2019**, *281* (March), 110–122. https://doi.org/10.1016/j.micromeso.2019.03.006.
